# Earlier Provision of Gastric Bypass Surgery in Canada Enhances Surgical Benefit and Leads to Cost and Comorbidity Reduction

**DOI:** 10.3389/fpubh.2020.00515

**Published:** 2020-09-30

**Authors:** Jason A. Davis, Rhodri Saunders

**Affiliations:** Coreva Scientific, Königswinter, Germany

**Keywords:** Roux-en-Y gastric bypass, obesity, diabetes, surgical delay, patient dropout, decision-analytic model, bariatric surgery, costs

## Abstract

**Background:** Effective provision of bariatric surgery for patients with obesity may be impeded by concerns of payers regarding costs or perceptions of patients who drop out of surgical programs after referral. Estimates of the cost and comorbidity impact of these inefficiencies in gastric bypass surgery in Canada are lacking but would aid in informing healthcare investment and resource allocation.

**Objectives:** To estimate total and relative public payer costs for surgery and comorbidities (diabetes, hypertension, and dyslipidemia) in a bariatric surgery population.

**Methods:** A decision analytic model for a 100-patient cohort in Canada (91% female, mean body mass index 49.2 kg/m^2^, 50% diabetes, 66% hypertension, 59% dyslipidemia). Costs include surgery, surgical complications, and comorbidities over the 10-year post-referral period. Results are calculated as medians and 95% credibility intervals (CrIs) for a pathway with surgery at 1 year (“improved”) compared with surgery at 3.5 years (“standard”). Sensitivity analyses were performed to test independent contributions to results of shorter wait time, better post-surgical weight loss, and randomly sampled cohort demographics.

**Results:** Compared to standard care, the improved path was associated with reduction in patient-years of treatment for each of the three comorbidities, corresponding to a reduction of $1.1 (0.68–1.6) million, or 34% (26-41%) of total costs. Comorbidity treatment costs were 9.0- and 4.7-fold greater than surgical costs for the standard and improved pathways, respectively. Relative to non-surgical bariatric care, earlier surgery was associated with earlier return on surgical investment and 2-fold reduction in risk of prevalence of each comorbidity compared to delayed surgery.

**Conclusions:** Comorbidity costs represent a greater burden to payers than the costs of gastric bypass surgery. Investments may be worthwhile to reduce wait times and dropout rates and improve post-surgical weight loss outcomes to save overall costs and reduce patient comorbidity prevalence.

## Introduction

As in other jurisdictions, Canada is experiencing an increasing burden of obesity and related comorbidities (1). This burden, in the form of chronic (type 2 diabetes, hypertension, or dyslipidemia, among others) or acute disease (stroke, heart failure), has a considerable and negative impact on patient health and on healthcare budgets. Structured bariatric management has been shown to be the best means of addressing obesity and related comorbidities. Patients in non-specialist care may achieve some initial improvements, but these often fade over time, leading to non-clinically significant weight loss (2), or weight gain (3). For many patients, the addition of bariatric surgery has been shown to yield significant improvements in weight loss and comorbidity remission compared to intensive medical management alone (4). Roux-en-Y gastric bypass (RYGB) and sleeve gastrectomy (SG) are the two most prominent forms of surgery provided in Canada (5).

Reviews of bariatric surgery in Canada have suggested the procedure is under-accessed and under-utilized as an effective strategy to manage patient weight and correspondingly, prevalence of obesity-related comorbidities (5). Numerous reports have acknowledged the issue of limited bariatric service capacity and its impact on patient health, including additional costs and mortality while awaiting surgery (6–10). After referral for surgery, some patients in Canada do not complete the program for various reasons including dropout or seeking surgery elsewhere. Recent studies in the province of Ontario have found significant predictors of non-completion of surgery to include male sex, increasing age, the occurrence of diabetes, longer wait times, and non-white race (11, 12).

For public payers such as the provincial healthcare systems in Canada, costs are a necessary consideration for the delivery of care and investments for potential system-wide improvements. A recent study of patients in the Ontario Bariatric Network, a centralized management system for all bariatric surgery referrals in the province, noted the potential negative cost impact of prolonged presurgical patient workup and its contribution to patient attrition (11). Other modeling studies based on published data from Canada have sought to quantify the cost burden associated with the current standard of care in comparison to an improved complete SG bariatric surgery care pathway in Canada (13), and focused on the post-surgical period for RYGB (14). These studies identified potential savings in costs and reduced patient comorbidity with realistic improvements to the presurgical wait time and post-surgical weight loss trajectories.

The present study is a decision analytic economic evaluation of the delivery and management of RYGB in the Canadian setting. In contrast to previous studies, the complete RYGB path from referral to a post-surgical time horizon is assessed, and the costs of surgery and surgical complications are incorporated in addition to the cost of treatment of diabetes, hypertension, and dyslipidemia. The cost and comorbidity treatment outcomes are quantified for an improved surgical care pathway (shorter wait time after referral and greater weight loss after surgery) compared to a Canadian standard care path. For additional context, the cost of comorbidity treatment is compared to the cost of surgery as well as the estimated risk of comorbidity prevalence for patients who do not undergo surgery. In the absence of suitable patient data to address unmet needs in Canadian bariatric surgical care, the estimates of the present study may provide a basis for discussions among policymakers regarding investments and with patients regarding comorbidity risk to investigate means of improving care for patients with obesity in Canada.

## Materials and Methods

This study is a decision analysis examining economic and patient outcomes. Prior to study commencement, the data protection officer of Coreva Scientific performed an assessment for risk of personal and/or identifiable data in compliance with the European General Data Protection Regulation. Ethical approval for this study and written informed consent from the participants of the study were not required in accordance with local legislation and national guidelines. It adheres to the Consolidated Health Economic Evaluation Reporting Standards (CHEERS).

### Post-referral Analysis

The primary outcome of the analysis was to assess costs from the perspective of a public payer for patients in a bariatric surgical care program in Canada. Informed by published data, the decision analysis estimates the differences in these costs between a standard care pathway (taken as the average Canadian bariatric surgical care pathway) and a pathway with improvements in shortened wait times and improved post-surgical weight loss. Costs include primary RYGB surgery and associated complications and comorbidities (diabetes, hypertension, and dyslipidemia) over a 10-year time horizon. The time horizon was chosen to allow capture of comparable post-surgical data for the standard care and improved care pathways, since surgery will occur at different times. Differences are expressed as costs (or comorbidity prevalence) in the improved path minus corresponding values in the standard care path.

### Pre-surgical Period

Few studies provide detailed data on patients during the presurgical period. The study of Padwal et al. (15) provides such data in the Canadian setting (the province of Alberta) from which parameters relevant to the study can be estimated. Patient demographics and baseline comorbidity prevalence (type 2 diabetes, hypertension, and dyslipidemia) are taken from the study (Table 1) and these values are broadly consistent with reports of comorbidity prevalence in other Canadian studies (Supplementary Materials, section 1, Supplementary Table 1). Incidence of comorbidity onset is modeled using data from patients who were waitlisted and some who were denied surgery in the US setting as the only identified source of data describing comorbidity evolution among bariatric surgery candidates (Supplementary Materials, section 1) (17).

**Table 1:** Model parameters.

**Parameter**	**References**	**Base case**	**Notes**
Age	Padwal et al. (15 )	43.6 ± 9.2 years	Canadian studies. Use population demographics of the waitlisted cohort (15 ), taken to most closely represent patients after referral before self-selection in population undergoing surgery may occur. Proportion of patients with diabetes whose disease may considered severe from reference (16 ) as surgical costs increase for this subpopulation.
BMI	Padwal et al. (15)	49.4 ± 8.2 kg/m^2^	
Female	Padwal et al. (15)	90.7 ± 2.4%	
Diabetes baseline	Padwal et al. (15)	50.0 ± 4.1%	
Proportion with severe diabetes	Doumouras et al. (16)	8.7 ± 2.8%	
Hypertension baseline	Padwal et al. (15)	66.0 ± 3.9%	
Dyslipidemia baseline	Padwal et al. (15)	59.3 ± 4.0%	
Diabetes incidence	Al Harakeh et al. (17)	3.0 ± 0.7%	Data include waitlisted RYGB patients and patients denied surgery in the American setting (17). Linear regression performed to determine an incidence rate for each to be used in presurgical and dropout patients. See Supplementary Materials, section 1.
Hypertension incidence	Al Harakeh et al. (17)	14.7 ± 8.2%	
Dyslipidemia incidence	Al Harakeh et al. (17)	3.6 ± 1.3%	
Dropout rate	Padwal et al. (15) Doumouras et al. (11)	6.3 ± 2.5% (improved) 19.6 ± 2.7% (standard) 22.9 ± 0.3% (Ontario)	These values are taken from a study in Alberta and used to represent the average Canadian standard of care since the surgical wait time described in the study more closely corresponds to the Canadian average 3.5 years. Values correspond to base case surgical times but are changed during sensitivity analyses where improved and standard care pathways have different surgical times. Data for Ontario, with an expedited care pathway compared to that reported in the Alberta study is used for a scenario analysis.
Time of surgery	N/A for model; Ontario Doumouras et al. (18)	1.0 year (improved) 3.5 years (standard) 1.0 year (Ontario)	Times are post-referral from primary to specialist care.
Cohort size	N/A	100 patients	Example cohort.
Discount rate	CADTH guidelines (19)	1.5%	4th edition guidelines of the Canadian Agency for Drugs and Technology in Health (CADTH).
Cost diabetes, year 1	Rosella et al. (20)	Male: $4,186 ± $628 Female: $4,141 ± $621	Ontario Base value uncertainty taken as ± 15%
Cost diabetes, year 2+	Rosella et al. (20)	Male: $854 ± $127 Female: $1,055 ± $128	Ontario Base value average costs years 2–8 in study.
Cost hypertension	Weaver et al. (21)	$2,163 ± $227	Canada wide.
Cost dyslipidemia	Conly et al. (22)	$79 ± $8	Alberta. Final value includes only laboratory costs for patients on statins minus costs for patient time and travel.
Cost gastric bypass surgery	CIHI Patient cost estimator^†^	$7,655 ± $1,046	Reported Canadian average from discharge database, uncertainty taken as standard deviation of individually-reported provincial gastric bypass costs.
Likelihood of complicated surgery	Supplementary Materials, section 2, Supplementary Table 4	10.2 ± 4.7%	Average rate of complicated bariatric surgery procedures (Supplementary Table 7).
Cost impact of complications on surgical costs	Doumouras et al. (23); Supplementary Materials, section 2	14.4 ± 15.5%	Complications and individual costs listed in Supplementary Tables 5, 6.
Cost impact of severe diabetes on surgery	Doumouras et al. (16)	54.1 ± 5.0%	Estimated impact of diabetes that is considered severe on the costs of surgery.
Cost impact of weight on comorbidity treatment	Alter et al. (24)	Obese 13.2% Overweight 5.0%	Independent effect of BMI to increase costs of treating comorbidity.

*BMI, body mass index. Costs in 2019 Canadian dollars, inflated from source data where necessary using Statistics Canada consumer price index data for health care items (Table 18-10-0005-01). CIHI, Canadian Institute for Health Information patient cost estimator, Canadian MIS Database, Discharge Abstract Database and Hospital Morbidity Database, Canadian Institute for Health Information, 2013–2014 to 2017–2018, data accessed 17 March 2020*.

Patient attrition during the wait for bariatric surgery has been identified as an issue in the care of patients with obesity (8, 11, 25). Among the studies analyzed for the present investigation, attrition data were identified for the provinces of Alberta and Ontario. The Alberta care pathway sees patients transition from a wait list to a weight loss program and ultimately surgery. In Ontario, an apparently expedited care pathway sees patients progress to surgery sooner, but with a higher dropout rate (Supplementary Materials, section 1, Supplementary Figure 1). The present model considers the Alberta care path since the wait times in the province are closer to the national average of 3.5 years than the wait times for Ontario, reported to be between 1 and 2 years [Supplementary Table 2 and references (18, 26)]. Outcomes for the Ontario care pathway (1 year wait, dropout rate 23%) are assessed in a scenario analysis. Patients who drop out are taken to continue to experience presurgical comorbidity incidence and do not spontaneously resolve.

Inclusion of a weight loss program in bariatric surgical programs across Canada is unknown. A previous study considered mild weight gain in the presurgical period (13), but as a more conservative approach in the present analysis, patients are taken to have stable weight on average; the sensitivity analysis includes possibilities of weight gain and weight loss during the presurgical period.

### Post-surgical Period

Considerable differences have been reported in weight loss trajectory after bariatric surgeries (27–29). One previous analysis has characterized outcomes in comorbidity resolution according to trajectory (27) and another considered the cost implications of comorbidity evolution for RYGB in the Canadian setting (14). Outcomes across Canadian studies of RYGB were assessed (Supplementary Materials, section 2, Supplementary Table 3) to determine trajectory groups from the study of Courcoulas et al. (27) that most closely resemble Canadian outcomes. To create a more realistic representation of likely clinical outcomes, patients are distributed across the two most closely matching trajectories in the standard care path and in the improved care pathway, patients are distributed across the next two best trajectory groups (according to improved weight loss, Supplementary Table 4).

### Model Design

A schematic of patient flow through the model is shown in Supplementary Image 1. Modeling parameters are presented in Table 1. As described above, demographics are taken from the Canadian study closest to Canadian average wait times (15) and applied to hypothetical cohorts of 100 patients who follow either the standard care or an improved care pathway. Standard care patients are taken to receive surgery at the Canadian average wait time of 3.5 years with average trajectory outcomes afterwards, and patients in the improved pathway receive surgery at 1 year with improved post-surgical weight loss trajectories. The Ontario scenario has patients with surgery at 1 year and standard Canadian post-surgical outcomes.

### Costs

Costs include surgery and comorbidities (diabetes, hypertension and dyslipidemia as three comorbidities that have been assessed according to post-RYGB trajectory) (27) Costs for complicated RYGB procedures (Supplementary Materials, section 3) were estimated from data in Ontario of incidence and cost of individual complications that included leaks and costs for readmissions (Supplementary Tables 5, 6). The risk of a complicated procedure was estimated from reported rates of complications after bariatric surgery in Canada (Supplementary Table 7). All costs were inflated to 2019 Canadian dollars using Statistics Canada consumer price index data for health care items (Table 18-10-0005-01). Costs were calculated separately for surgery and for comorbidities to determine the relative contributions of each to total care costs for patients in the (RYGB) bariatric surgery care pathway.

### Sensitivity Analyses

The main analysis incorporates sensitivity analyses by sampling within uncertainties associated with model parameters (10,000 replicates of 100-patient cohorts following each pathway) to encompass a broad range of potential patient characteristics and outcomes. Additional sensitivity analyses were performed to assess the independent effects of improvements to the wait time (shorter time from referral to surgery) and post-surgical weight loss. Surgical wait times in the improved pathway were varied from 6 months to 2.5 years for comparison with standard care wait times of 2.5–5 years, as have been seen in Canada (Supplementary Table 2). Outcomes were also assessed with changes to wait time with no improvement in post-surgical trajectory. A further analysis compared outcomes of the surgical care path with non-surgical care (all patients drop out at the time when surgery occurs for surgical patients). Costs were compared to determine the break-even point (return on surgical investment) where total cumulative costs for each would be the same.

### Statistical Reporting

Non-parametric statistics were used in the analyses, reporting results as medians and 95% credibility intervals (95% CrI) from repeated sampling (10,000 replicates for main analysis, 2,500 replicates for combinations in sensitivity analyses). Inference testing was not performed, and as such, claims regarding clinical relevance of outcomes are not made. Credibility intervals that do not overlap, difference intervals that do not include 0 (no difference), and ratio intervals that do not include 1 (no difference) may be considered indicative of differences that warrant further investigation. All calculations performed using the R statistical language (version 4.0).

## Results

**Figure 1:**
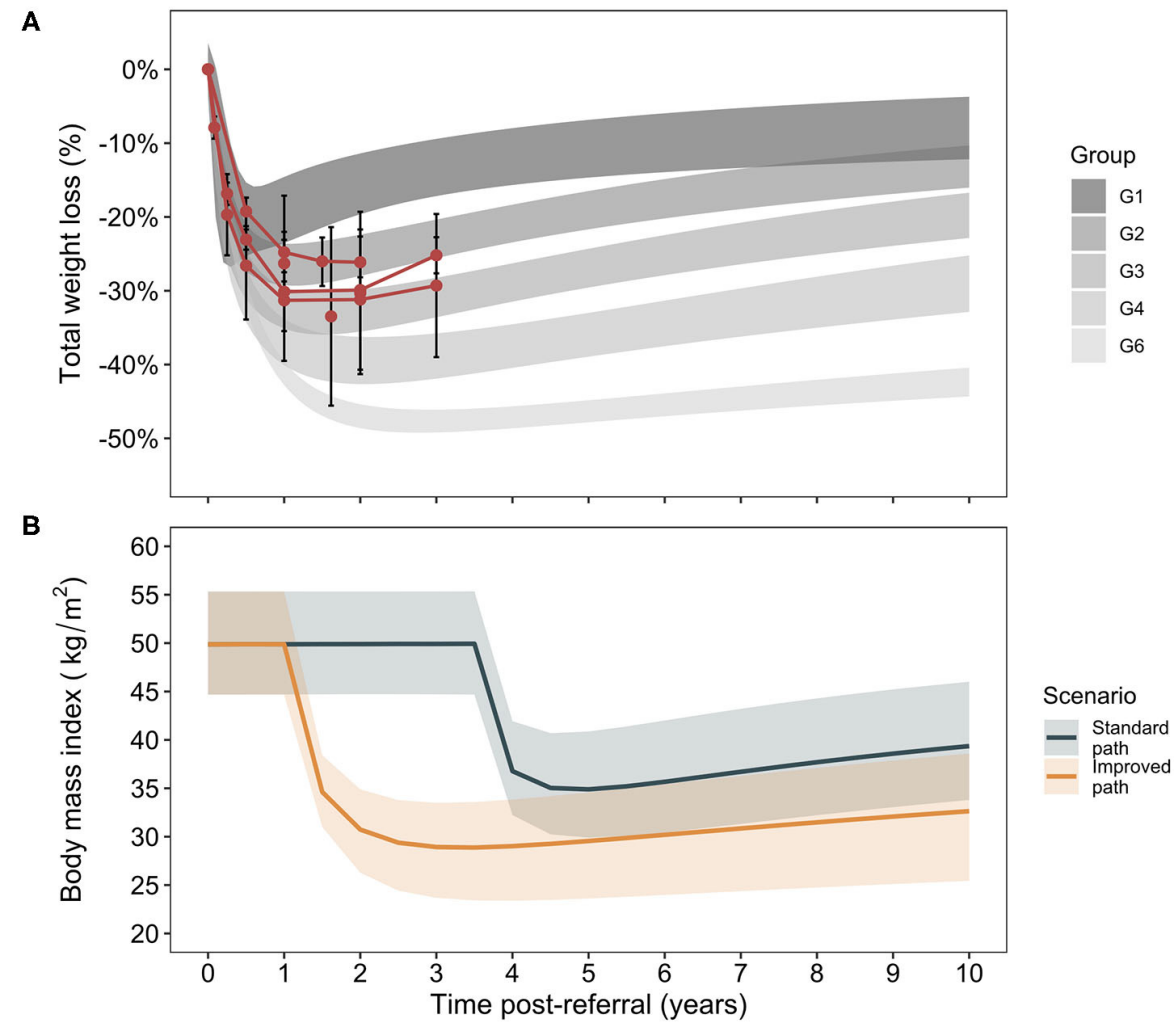
Trajectory analysis of weight loss outcomes after Roux-en-Y gastric bypass. Data identified for weight loss outcomes after Roux-en-Y gastric bypass surgery in Canada (Supplementary Table 3, means and standard deviations) are plotted against the trajectory groups for patients after RYGB as reported in an analysis of post-RYGB total weight loss trajectories [95% CrIs, **(A)**] (27). For clarity, a group 5 in the original analysis that demonstrated atypical weight loss patterns is not shown, as it has been excluded from the present analysis. For the present model, Canadian outcomes are associated with trajectories 2 and 3 and the improved scenario of increased weight loss is comprised of trajectories 4 and 6 **(B)**. The cohort trajectories in terms of body mass index evolution over time post-referral in the two care pathways is shown. Lines correspond to weighted medians (for trajectories comprising the given scenario) and bands to 95% CrIs.

The decision analysis considers comorbidity and cost outcomes between a standard care and improved care RYGB bariatric surgery program in the Canadian setting. An overview of study results for primary and sensitivity analyses in the main and supplementary materials is shown in Supplementary Image 2. To determine the potential for improvement after surgery, Canadian RYGB outcomes were plotted against trajectories previously reported (Figure 1) (27). Canadian total weight loss results largely overlap with the central trajectories of the previous analysis and these were thus taken to represent the Canadian standard of care with the improved care pathway indicated by a shift to the next best set of trajectories (Supplementary Table 4). Outcome trajectories for the current model population [age 43.6 ± 9.2 years, body mass index [BMI] 49.4 ± 8.2 kg/m^2^, 90.7% female] show the broad range of weight loss outcomes (as change in BMI) assessed in the current study with uncertainty intervals and a modest improvement in final BMI in the improved care pathway.

Surgical costs are incurred once during the post-referral period at the time of surgery (3.5 years in the standard care pathway and at 1 year in the improved and Ontario care pathways). Comorbidity costs vary according to the evolution of prevalence of diabetes, hypertension, and dyslipidemia. Prevalence is comprised of new cases (presurgical incidence rates in dropout patients and post-surgical rates for surgical patients), cases present at baseline who did not achieve remission, and cases that change between remission and relapse post-RYGB. Cumulatively fewer cases of comorbidities are associated with the improved vs. standard care pathway (Supplementary Figure 2), indicating reductions of 200 (95% CrI 138–258), 246 (95% CrI 95–309), and 199 (95% CrI 123–262) patient-years of treatment for diabetes, hypertension, and dyslipidemia, respectively, for a 100-patient cohort over 10 years (Supplementary Materials, section 4, Supplementary Figure 2).

**Figure 2:**
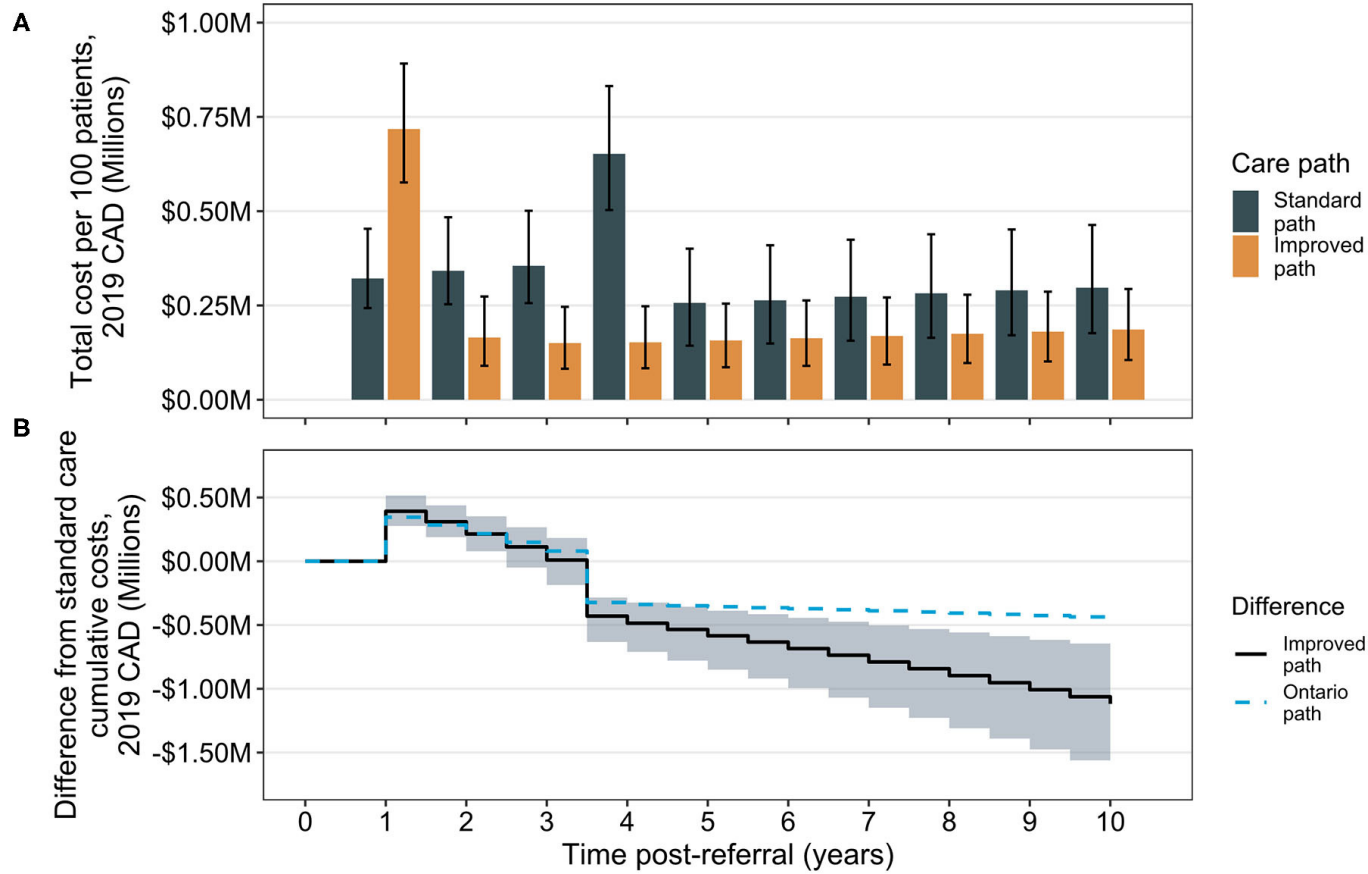
Total and cumulative cost differences between the standard care and improved bariatric surgical care pathways. Total annual costs (sum of surgical and comorbidity costs) are shown by year over the 10-year time horizon. Bars indicate median totals and error bars are 95% CrIs **(A)**. The cumulative difference is calculated as the annual total for the improved pathway, minus the total in the standard care pathway; the solid line indicates the median difference and the shaded region the 95% CrI about the median **(B)**. For comparison, results from analysis of cumulative differences for the Ontario pathway are shown (median only), for which surgery occurs at the same time as in the improved path (1 year post-referral) but after surgery, patients experience the standard path scenario of weight loss trajectories. CAD, Canadian dollars.

The associated total annual costs of treatment differ considerably depending on time after referral in the two care pathways (Figure 2). At 1 year post-referral, the improved care group is associated with an increase in costs compared to the standard care group due to the occurrence of surgery. Costs remain higher in this group until the standard care group experiences an increase in annual costs attributed to its performing surgeries. Comparison of cumulative costs suggests a break-even inflection point at ~3.5 years, the mean time of surgery in the standard care group, where total cumulative costs in the improved care group are expected to be lower than those in the standard care group (Figure 2). For comparison, data from Ontario are shown overlaid (without corresponding credibility intervals for clarity). The divergence in cost reduction associated with the Ontario scenario from the median observed in the modeled improved care pathway group is due to a combination of the higher dropout rate in the Ontario pathway (meaning fewer patients can achieve comorbidity remission from surgery) and due to the improved post-surgical weight trajectory in the improved pathway. The Ontario result suggests wait time alone is not the sole influence on overall costs, but that dropout rate and post-surgical trajectory may also be contributors.

**Figure 3:**
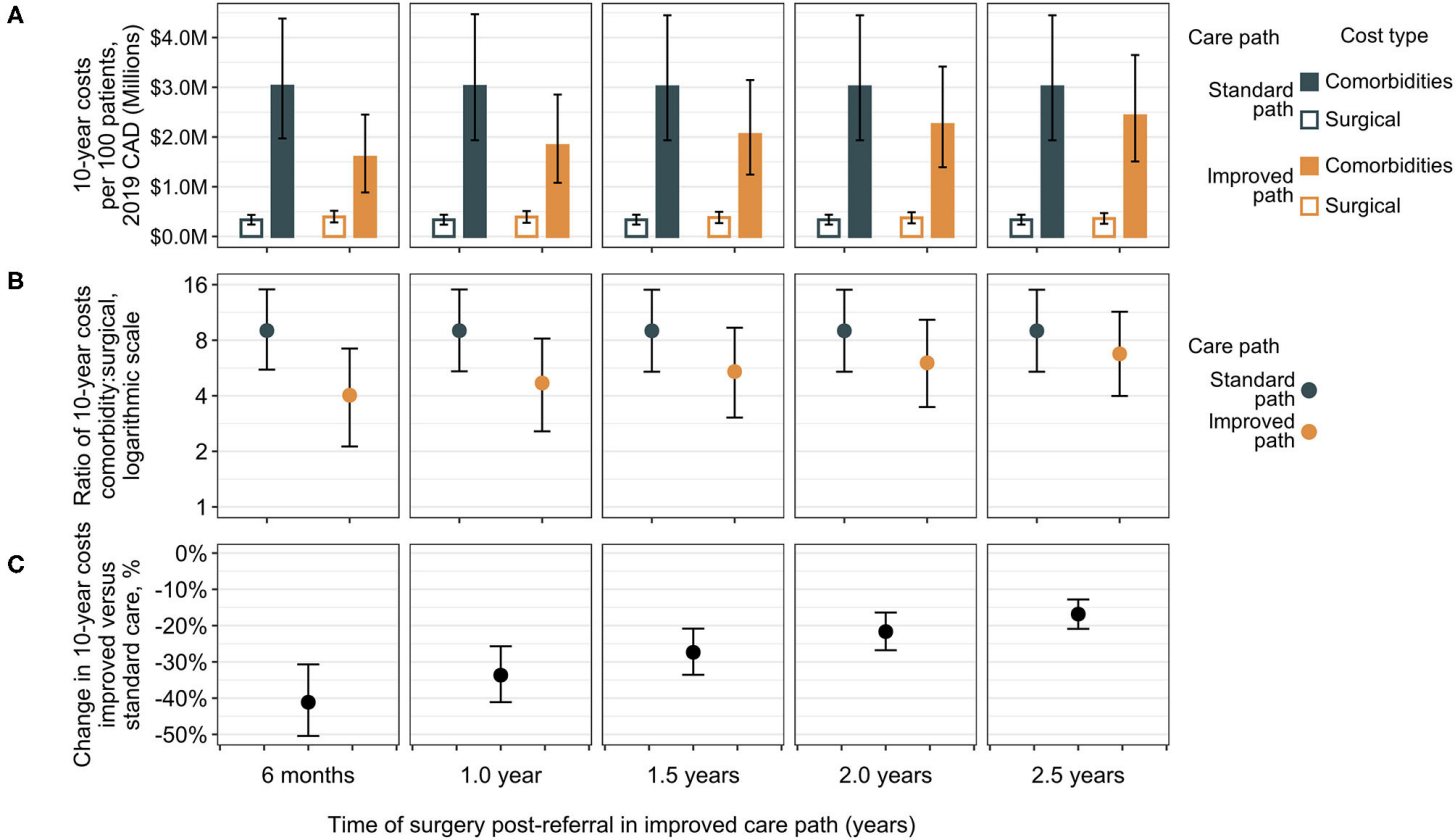
Sensitivity analysis of surgical vs. comorbidity cost outcomes. Total 10-year costs are shown separately for surgical costs and comorbidity costs for differing improvements in time of surgical delivery as compared to the standard care pathway with surgery at 3.5 years post-referral [medians with error bars indicating 95% CrIs, **(A)**]. Surgical costs are lower in the standard care group compared to the improved group, but the ratio of total comorbidity costs to surgical expenditure is higher [medians with 95% CrIs, **(B)**]. The difference in total costs (surgical plus comorbidity) indicates a decrease in total 10-year bariatric surgical patient costs in the improved care pathway vs. the standard care pathway **(C)**. The largest differences (greatest cost reductions) are associated with the earliest delivery of surgery in the improved care pathway at 6 months and decrease as the improved path surgery approaches the standard care surgery wait time, but at all intervals of improvement (from 3 to 1 year earlier surgery), the corresponding 95% CrIs of change in total costs are exclusive of zero. CAD, Canadian dollars.

The total costs of care for a patient cohort in the RYGB bariatric care pathway in the present analysis are comprised of surgical costs (including complications) and comorbidity costs. These can be calculated separately and assessed to test the effect of varying improvements to the time of surgical delivery in the improved care pathway via sensitivity analysis (Figure 3). Results are shown for improved surgery from 6 months to 2.5 years compared with the standard care surgery at 3.5 years. In each case, the standard care pathway is associated with lower total surgical costs as a combination of discounting and patient dropout, since fewer patients will undergo surgery due to dropout. Over a 10-year period, however, considerably higher costs to public payers are associated with comorbidities than for surgeries, regardless of when surgery occurs, suggesting that surgery and its complications represent a relatively smaller proportion of care costs for patients with obesity in the surgical care pathway. The standard care pathway is associated with higher ratios of comorbidity treatment costs to surgical costs compared to ratios in the improved pathway, but the difference diminishes as the improved time of surgery approaches standard care (Figure 3). Considering total costs (surgical and comorbidity together), the improved pathway is associated with median percent reductions from 41% (95% CrI 31–50%) if the improved path delivers surgery 6 months post-referral, to 17% (95% CrI 13–21%) if surgery occurs at 2.5 years in the improved pathway (Figure 3). Similar results were observed across a broader range of standard care wait times with various improvements in shorter wait lists (Supplementary Figure 3, Supplementary Table 8).

Further sensitivity analyses tested the robustness and independence of wait time and post-surgical weight loss improvements to the observed reduction in costs. Delivery of surgery at the same time in both care pathways, but retaining the post-surgical improvement revealed that the associated benefits of comorbidity cases and cost reduction for the improved pathway decreased with longer surgical delays. Surgery at 6 months post-referral in both pathways was associated with a 21% (95% CrI 14–27%) reduction in total 10-year costs, but this reduction decreased to 5.0% (95% CrI 1.8–7.9%) if surgery were delayed to 5 years (Supplementary Figure 4). If both paths achieved the standard care post-surgical weight loss outcomes but surgery were brought forward, total costs (Supplementary Figure 5) and percentage reductions (Supplementary Table 9) show the same pattern as when both wait time reduction and increased post-surgical weight reduction are included in the improved path. The magnitude of the change is, however, considerably decreased. Compared to standard care surgery at 3.5 years, an improved delivery of surgery at 6 months was associated with a 26% (95% CrI 13–36%) reduction, decreasing to 5.8% (95% CrI 2.5–9.8%) if surgery occurs at 2.5 years.

**Figure 4:**
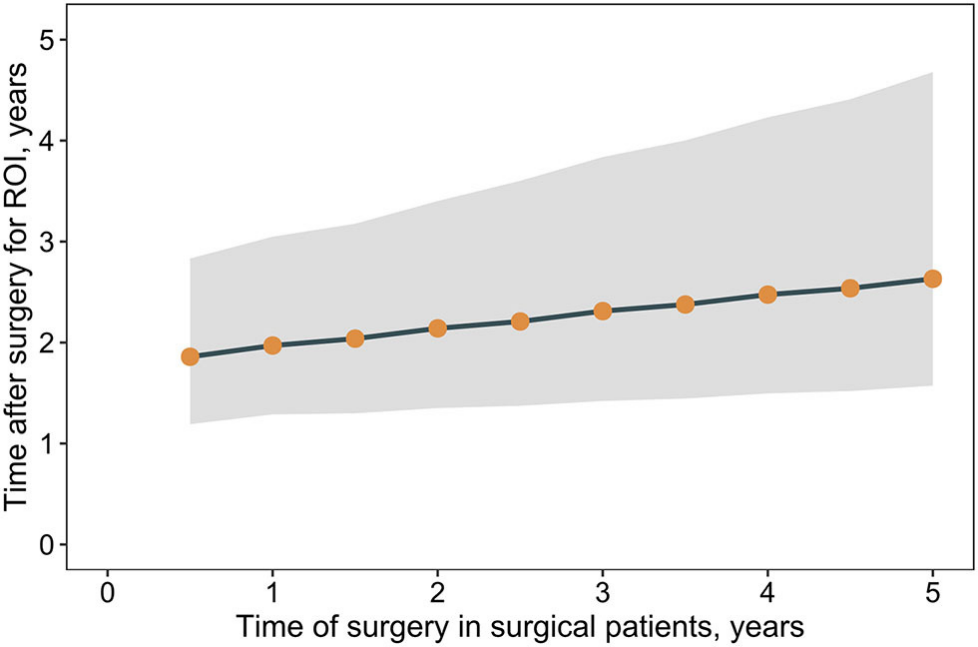
Return on surgical investment vs. non-surgical care vs. time of surgery. Shown are the median time post-surgery for total costs in a patient cohort undergoing surgery (surgical and comorbidity costs) to equal total costs for a patient cohort that does not undergo surgery (comorbidity costs only), that is, the return on surgical investment. Time post-surgery estimated from linear regression cumulative cost differences between the surgical and non-surgical care pathways. Points indicate medians and the band corresponds to the 95% CrI.

**Figure 5:**
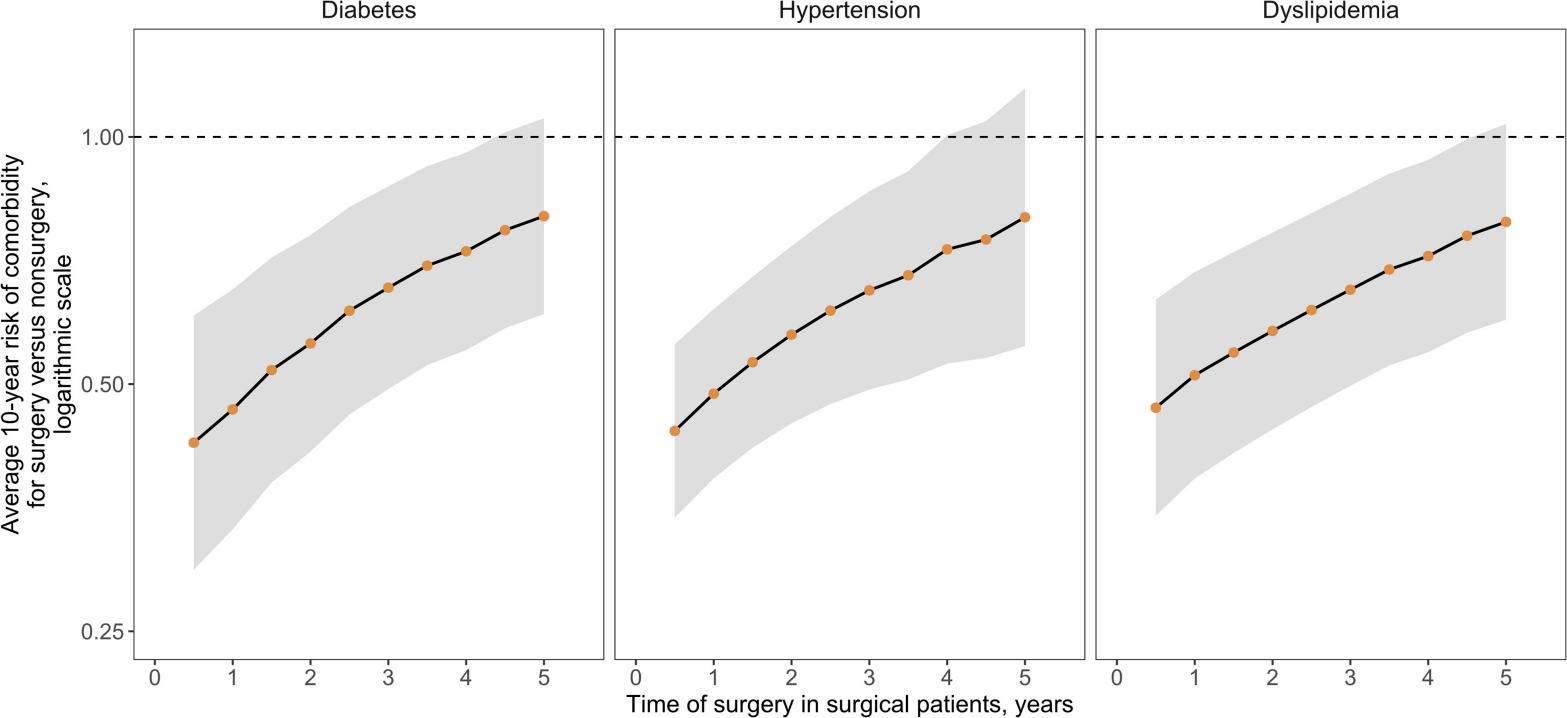
Relative risk of comorbidity prevalence for surgical vs. non-surgical patients. Disease prevalence over the 10-year time horizon was used to calculate relative risk (points) and 95% Confidence Intervals (CIs, normal distribution statistics) for presence of the indicated comorbidity in the surgical vs. non-surgical cohort depending on the time of surgical delivery.

The results suggest an initial sharp increase in costs is associated with the provision of RYGB but that over time, costs of comorbidity treatment may far outstrip the costs of surgery. Analyses were undertaken to estimate when, after surgery, a return on the surgical investment may occur, as determined by the break-even point where total cumulative costs in the surgical pathway are the same as those in the non-surgical pathway. The time taken to achieve a return on investment is trends toward increases as the time of surgery is delayed (Figure 4). A similar association between outcomes and surgical delays is reflected in the relative risk of comorbidity prevalence in surgical vs. non-surgical patients as the time of surgery is delayed (Figure 5). For each comorbidity assessed, the risk of having disease is over 2-fold lower when surgery is delivered 6 months after referral (relative risk of diabetes 0.424, hypertension 0.439, and dyslipidemia 0.468) and the corresponding 95% confidence intervals do not include 1.0. If surgery is delayed to 5 years, the risk in comorbidity prevalence increases (diabetes 0.801, hypertension 0.798, dyslipidemia 0.788) and the intervals include 1.0, suggesting that surgery on average will still decrease the risk of having a comorbidity, but to a lesser degree than if surgery had been delivered sooner. Note that these relative risks apply for the baseline disease prevalence in the current analysis (diabetes 50%, hypertension 66%, and dyslipidemia 59%) and would be expected to change for different baseline demographics. Sensitivity analysis where the surgical patients achieve standard care post-surgical weight trajectories revealed a similar trend, but the loss of potential significance of the reduction of relative risk occurs earlier (Supplementary Figure 7).

## Discussion

For the patient cohorts considered, the analysis suggests that improvements to the RYGB bariatric care pathway in Canada could result in considerable reduction of burden of comorbidity related to obesity and of public-payer costs. Sensitivity analyses indicated that these reductions were comprised of independent contributions from reducing the wait time for surgery and from improving post-surgical weight trajectories for increased weight loss. The results for the country on average can be considered against those specific to the province of Ontario, where the same improvement to surgical wait time has been achieved as proposed in the present model. While the Ontario results indicate better cost outcomes compared to the Canadian average, there may yet be room for improvement as authors have noted issues with patient dropout (11, 25), and weight loss in the post-surgical period overlaps with the middle to lower weight loss trajectories according to a cohort analysis of RYGB patients in the United States (27).

A previous analysis considered cost and disease treatment outcomes in Canada for SG surgery (13). While SG appears to be increasing in use around the world, including in Canada (5), RYGB continues to be a widely applied surgery in the Canadian setting, although its application varies widely by province. In 2014, among the provinces with the highest bariatric surgery volume, RYGB accounted for 84% of 2,833 surgeries in Ontario, 13% of 2,411 surgeries in Quebec and 48% of 540 surgeries in Alberta (6). Separate consideration of outcomes after RYGB is thus warranted, given the different effects of comorbidity resolution achieved after RYGB compared to SG.

Although this analysis was performed in the Canadian setting using Canadian costs, the results are potentially applicable to other settings where delays to provision of surgery or suboptimal post-surgical weight loss outcomes may occur. Expressed as relative changes in total costs, the improvements to the surgical care pathway in the base case scenario were associated with about a 34% reduction in total costs over 10 years even with the inclusion of surgical costs. The sensitivity analyses performed provide outcomes for a range of improvements for consideration by other localities. The precise difference in costs will in part depend on the relative cost of comorbidity treatment to the cost of surgery and as a guide, the base cost of surgery in this analysis was found to be approximately the same as the annual cost of an incident case of diabetes plus 1 year of hypertension treatment. Any healthcare system with similar proportions, or where comorbidity costs are higher still relative to surgery may be expected to see similar results. The result suggests that for any setting where surgical cost is a consideration for access to bariatric surgery, these costs should potentially be considered in the context of how much will be spent in treatment of related comorbidities over the 10-year post-referral period. The return on surgical investment considering multiple scenarios of surgery delivery time and improved weight loss outcomes indicates that within 3 years of surgery, total costs will be equivalent for a non-surgical cohort, but thereafter, costs in the surgical cohort will be lower.

A consistent observation throughout these analyses was the impact of timing of surgery on outcomes. The observed improvements in cost and burden outcomes diminished as the time of surgery was delayed. For patients, this effect is further manifest in the diminished comorbidity risk reduction for delayed surgery. Such information may be relevant for patients who have self-selected out of the bariatric surgery care program, given that future reconsideration will still, such that if they reconsider and reenter the program later, surgery will still be beneficial, but the impact on reducing the risk of comorbidity development may be lower.

Achieving a goal of reducing wait times across Canada toward those seen in Ontario will be influenced by multiple factors. The expedited pathway in Ontario sees earlier surgery but a higher dropout rate (11, 25, 26) compared to the Alberta pathway used in this study as the Canadian average (15). A key difference between the two is the inclusion of a weight management program in the Alberta pathway, but whether the addition of weight management or a higher degree of patient engagement is responsible for higher retention is unclear. In other settings, the insurance-mandated requirement of successful completion of presurgical weight loss programs as a prerequisite for surgery has not been associated with improved post-surgical outcomes (30), and a position statement from the American Society for Metabolic and Bariatric Surgery notes that with a lack of data from randomized controlled trials, this practice leads to unnecessary delays and the progression of life-threatening comorbid conditions (31).

Patient non-completion of surgery is another potential area to address. Predictors of patient attrition have been investigated (11, 25), but the reasons are unclear. Non-completion for many patients is self-directed, rather than physician-advised, and the patients no longer engage with the bariatric surgery program to explain why they have left (25). That the presence of diabetes is a predictor of non-completion is of concern, as these patients may especially benefit from surgical intervention for improved chance of remission of diabetes. The connection between non-white race and non-completion of surgery or even access to care also requires investigation, as this effect is not limited to the largely private payer systems of the United States (32, 33) but also has been observed in public payer systems as in Canada and elsewhere (12, 34).

The estimates generated in this study are based on sourced, relevant data from the Canadian setting, but as a model, assumptions are required for contributors that are unknown. The average standard care path taken from the Alberta provincial setting may not apply to every province. The experience and related costs of dropout patients are also unknown. Here, dropout patients are assumed to begin mild weight gain and have baseline, presurgical comorbidity incidence but it is not known whether this state would remain true over the 10-year time horizon. In the absence of data, the model cannot make predictions regarding additional interventions, including reentry to the surgical care pathway, or costs these patients may incur. The model also focuses on three main comorbidities (diabetes, hypertension, and dyslipidemia) as these were comorbidities for which outcome evolution data over 7 years post-RYGB surgery were available stratified by post-RYGB weight trajectory (27). The burden of other comorbidities, such as micro- and macrovascular disease, often associated with obesity, was not included. Since comorbidities were treated independently, multimorbidity and the associated increased risk of outcomes such as major cardiovascular events including stroke or myocardial infarction cardiovascular events, could also not be assessed. Any of these conditions could add considerably to the patient and public payer burden. That savings in costs were associated with the improved vs. standard care pathway, and for surgery vs. no surgery when including only these three comorbidities supports the conservative nature of the analysis. The improvements in wait time and post-surgical trajectory assessed are hypothetical, but within reason. Surgical delivery at 1 year post-referral occurs in the Ontario care pathway (11, 25, 26), and there is considerable overlap in the weight trajectories of the two care pathways (Figure 1) demonstrating non-significance of the weight loss improvements under consideration.

More real-world data on patient experience pre- and post-RYGB are required. In the post-surgical period, results are typically reported in aggregate for a cohort, but it is known that individual patients will experience different trajectories and different rates of comorbidity remission. Presurgically, more needs to be known regarding how comorbidities evolve and how to reduce patient attrition and wait times. In the absence of such data, model studies and associated sensitivity analyses provide a means of evaluating the potential impact of changes or improvements to healthcare delivery. Whether the improvements to RYGB surgical care for patients with obesity in the present study are feasible is subject to local resources and healthcare priorities within Canada or elsewhere. The results provide an estimate of excess burden that may be associated with current bariatric surgical care pathway inefficiencies specific to RYGB and these estimates may provide a basis to inform investments to improve care, especially within the context of other non-bariatric surgery healthcare expenditures.

## Data Availability Statement

The raw data supporting the conclusions of this article will be made available by the authors, without undue reservation.

## Ethics Statement

Ethical review and approval was not required for the study on human participants in accordance with the local legislation and institutional requirements. Written informed consent for participation was not required for this study in accordance with the national legislation and the institutional requirements.

## Author Contributions

JD performed the literature searches, data and statistical analyses, interpreted results, and wrote and revised the manuscript. RS contributed to the data interpretation and writing and revision of the manuscript. All authors contributed to the article and approved the submitted version.

## Conflict of Interest

JD and RS are employees of Coreva Scientific and have performed remunerated data analysis and consultancy work for Medtronic plc outside of the present work. JD and RS received funding in connection with the current study.
