# Supplementary material for: Earlier Provision of Gastric Bypass Surgery in Canada Enhances Surgical Benefit and Leads to Cost and Comorbidity Reduction
Source: Front Public Health. 2020 Sep 30;8:515. doi: 10.3389/fpubh.2020.00515 (PMC7554569; doi:10.3389/fpubh.2020.00515)
Supplement: Supplementary file 1 [file Data_Sheet_1.PDF]

Supplementary Material

1 Presurgical patient demographics and dropout in Canada

1.1 Patient demographics

Patients who are indicated for bariatric surgery often present with comorbidities at baseline. Indeed, the presence of comorbidity is often a prerequisite for surgical eligibility for patients with body mass index (BMI) of 35 to 40 kg/m^2. The study of Padwal et al., 2014 has been used for baseline comorbidity prevalence in the present model since the study presents data for waitlisted patients. These patients would be expected to most closely resemble the postreferral patients in the present study model. For comparison, other studies in the Canadian setting were examined for reported comorbidity prevalence at baseline (*Table S1*). The prevalence of diabetes (50%), hypertension (66%) and dyslipidemia (59%) are somewhat higher than the weighted provincial averages for these comorbidities (35%, 51%, and 32%, respectively) and for other provinces such as Ontario for which the most data are available (35%, 44%, and 3%, respectively). The values are still taken as reasonable, however, given that these demographics correspond to patients in the study on which the dropout data and care pathway are being modeled, and since they provide data on waitlisted patients. In Ontario, the occurrence of diabetes has been reported to be an independent predictor of surgical dropout.(1) In the Padwal et al., 2014 study here, the prevalence of diabetes in the waitlisted population (50%) was higher than in the population who ultimately underwent surgery (44.7%).(2) Assuming these patients did not achieve comorbidity resolution due to waitlisting or medical management, these data are consistent with the suggestion that patients with comorbidities may self-select out of the surgical pathway.

1.2 Presurgical weight and comorbidity evolution

According to the description of the bariatric surgery care pathway in Alberta, after referral, patients are wait-listed after which they are enrolled in a weight management program (Weight Wise).(2) Published results suggest that, on average, patients achieve a degree of weight loss. Although this path is being used to represent a Canadian average, it is unknown whether all provinces have such a program. Ontario is a notable example, where patients are described to receive nutritional support, but without indication of a formalized program.(3, 4) Weight change for the present model was taken to be stable (that is, no change during the wait period) in the present model, with sensitivity analyses including the possibility of a cohort on average gaining or losing weight.

Limited data are available regarding comorbidity evolution in the wait-listed bariatric surgery population. The data of Al Harakeh et al.(5) reported comorbidity incidence for diabetes, hypertension, and dyslipidemia for patients awaiting and who were denied bariatric surgery. A previous analysis modeled these data using power law functions,(6) which assumes dynamic change (in this case increase) in comorbidity incidence over time. As a more conservative approach for the present analysis, these data are modeled using linear regression and a constant incidence is applied that does not increase over time. This decision was taken to apply presurgical incidence rates to dropout patients after the decision point where patients are counted as dropping out or continuing with surgery. Dropout patients continue with the presurgical comorbidity incidence rate while surgical patients are subject to postsurgical remission, relapse, and incidence according to postsurgical trajectory. Extrapolation of the power models over the 10-year time horizon for the dropout patients would result in rates of 20-50%

depending on the comorbidity by 5 years postreferral100% within a few years, and these were deemed unrealistically high.

Linear regression of the presurgical (and denied surgery) data of Al Harakeh et al.(5) considered over the first year (time 0 to time 1 year postreferral) yields constant incidence rates of 3.0% ± 0.7% for diabetes, 14.8% ± 8.2% for hypertension, and 3.6% ± 1.3% for dyslipidemia.

1.3 Patient dropout

Not all patients indicated for surgery will undergo the procedure. As part of the present study, analysis was performed to model the dropout rate akin to a survival analysis as a function of time waiting. Studies in the Canadian setting were identified that provided relevant data for the province of Alberta(2) and Ontario.(3, 4)

The study of Padwal et al.,(2) set in Alberta examined 3 phases of the bariatric care pathway: waitlisted patients, those undergoing medical management, and those undergoing surgery. Patient attrition was reported at each stage and these data were combined with the average length of time in each stage to estimate patient retention rates over the entire presurgical period (*Figure S1*). Of the total 92 patients lost to attrition, 78 (85%) were lost due to dropout while the remainder left due to pregnancy, death, or opting for out-of-province surgery.(2) Patient counts reported at each time were therefore scaled by 85% to consider only dropout patients.

Data were fit using an exponential relationship. An earlier study in Alberta that examined patient outcomes reported a similar care pathway, where patients prior to surgery participate in the "Weight wise" medically-managed weight loss program.(7) After referral, patients are waitlisted, then enter the Weight wise program for a fixed duration, after which they may remain with the program or crossover to surgery.

The studies of Diamant et al.,(3, 4) for Ontario describe an expedited surgical care pathway. Patients in Ontario receive care under the auspices of the centralized Ontario Bariatric Network, a system of regional centers to manage bariatric care in the province. After referral, patients attend an orientation session, and progress through nursing, social work, nutritional, psychological and surgical assessments prior to surgery.(4) In contrast to the Alberta system, there is no apparent medically-managed weight loss stage. Patient attrition appears considerably higher with around 50% loss by the 1 year average time to surgery (Figure S1). A more recent study,(1) found time to be an independent predictor of attrition, with the odds of dropping out increasing by 5% for each month of waiting up to the average 1 year surgical time. Overall dropout was 23%, but the timing of dropout was unknown.

For the present study, the Alberta data are taken to model the average Canadian care pathway. The care pathway for each province is unknown, however, the average wait time for Alberta is closer to the national average than the wait time associated with the expedited pathway in Ontario (Table S2).(8) Analysis using the Ontario care pathway with a mean time to surgery of 1.0 year and assigned the more recent(1) dropout rate of 23%.

2 Weight loss outcomes of Roux-en-Y gastric bypass surgery in Canada

The data of Courcoulas et al.,(9) 2018 provide a detailed stratification of outcomes by weight loss trajectory for patients after Roux-en-Y gastric bypass (RYGB) surgery. To identify which trajectory or

trajectories from the Courcoulas et al. cohort would best represent reported outcomes in Canada, a review of the literature was performed to identify relevant studies in the Canadian setting. The focus was on those that reported outcomes specific to RYGB, separately from other types of surgery. that reported outcomes for RYGB surgery.

As seen in the main text (Figure 1), results from Canada mostly overlap with trajectory groups 2 and 3 (listed in Table S3). To simulate more realistic patient outcomes, the standard care scenario in this study considers patients after RYGB in Canada to be distributed among these weight loss trajectories by redistribution of proportions reported in the original study by Courcoulas et al.,(9) 2018 (Table S4). The decision analysis of the current study assesses the outcomes for a reasonable improvement in weight loss, whereby patients are redistributed among the top two trajectories (groups 4 and 6) when considering weight loss. Group 5 was excluded from the present analysis since it represents a small proportion of the original study group (6.1%) who experienced an unusual trajectory with low early weight loss that later accelerates and demonstrates increasing weight loss even at 7 years of follow-up.(9) A separate study of these patients may be beneficial to identify factors that may aid in recovery of the procedure (patients who demonstrate early, poorer weight loss who can be converted into high and continuing weight loss) but such an analysis is beyond the scope of the present study.

3 Impact of surgical complications on costs of Roux-en-Y gastric bypass in Canada

A base surgical cost is reported in the main text of $7,655 (2019 Canadian dollars). Complications arising from surgery add to costs, but the exact impact on costs will depend on the nature of the complication and the cost of its management (*Table S5*).(10) Using data from Ontario, the average additional cost due to complications was determined as the average incidence of each complication multiplied by the associated cost to yield an average cost due to complications (*Table S5*).

Surgical costs for RYGB in Ontario have been reported over years 2009–2012 (*Table S6*). Given the uncertainty indicated (for example, $13,253 ± $47,539 for 2009–2010), these costs likely include complication costs. To determine the cost impact of complications, the cost of an uncomplicated procedure was estimated by subtracting the average cost burden (complications and readmissions, *Table S5*) from this average and expressing the additional burden as a percentage (*Table S6*). Note that since the uncertainty is larger than the value, the sensitivity analysis that samples over this range will include the possibility of surgical costs decreasing.

A brief survey of the literature was performed to estimate the probability of complicated RYGB procedures (*Table S7*). More recent studies in Canada suggest complication rates associated with surgery (within 30 days) tend to be lower (under 10%) compared to other studies in the United States (15.4%)(11) and earlier studies in Canada (11.9%).(12) As the focus in the present study regarding surgical costs is focused on the surgical period, those with longer data periods (complications that occur 1-2 years after surgery) are not included. A conservative 10.2% probability of a complicated RYGB procedure is therefore estimated for use in the model.

4 Raw totals and relative change of comorbidity treatment and costs between standard of care and improved pathways

In the main text (Figure 2), total cost outcomes are shown for 100-patient cohorts following either the standard care pathway (surgery at 3.5 years postreferral, standard care postsurgical weight trajectories) or the improved care pathway (surgery at 1 year postreferral, improved postsurgical weight trajectories). Total costs are the sum of surgical costs and cost of treating comorbidities (diabetes, hypertension, and dyslipidemia). For further illustration of potential differences in treatment pathway, the total annual and cumulative difference in the number of cases treated (in patient-years) was determined for each of the comorbidities in the present study (*Figure S2*).

5 Sensitivity analyses

Sensitivity analyses were performed to test the robustness of the analyses performed in the base case. The main text (Figure 3) presents surgical and comorbidity total costs, ratio of comorbidity to surgical costs, and the overall cost savings for varying degrees of improved time of surgical delivery in the improved care path versus the standard care path, whose surgery occurs at 3.5 years post referral.

Provincial wait times for bariatric surgery vary (*Table S2*). To account for different starting standard care pathways and to allow assessment of differing degrees of wait list shortening according to provincial resources, multiple combinations of standard care and improved care surgical wait times were analyzed for total costs (*Figure S3*) and for the corresponding percentage change in total costs (*Table S8*). Note that as in the main analysis, these cost differences include postsurgical weight trajectory improvement in the improved care pathway versus standard care.

Results suggest an association between the improved care versus standard care pathway and reductions in cases of comorbidities treated and corresponding costs. In *Table S8*, a reduction is still observed where both the standard care and improved care pathways deliver surgery at 2.5 years post referral due to the contribution of postsurgical weight trajectory improvement. To determine the effect of time post referral of surgical delivery on the reduction in total costs due to improvement in postsurgical trajectory, a sensitivity analysis was performed in which both care pathways receive surgery at the same time post referral, but the improved care pathway experiences the scenario of greater weight loss trajectories after surgery (*Figure S4*). When both paths have surgery at the same time, the benefit of improving the postsurgical weight trajectory decreases from a reduction of 20.9%, 95% CrI [14.4% to 27.3%] when surgery occurs 6 months post referral to a reduction of 5.0%, 95% CrI [1.8% to 7.9%] if surgery is delayed to 5 years post referral.

Similarly, to separate the effect of postsurgical trajectory on cost outcomes, the sensitivity analysis of varying time of surgical delivery in the standard care and improved care pathways of *Figure S3* and *Table S8* were repeated for the scenario where both care pathways experience standard care weight loss trajectories after surgery. Total costs total costs (*Figure S5*) and the corresponding percentage change in total costs (*Table S9*) were calculated, revealing lesser reductions in expenditure when the improvement in postsurgical weight trajectory is removed. As an additional confirmation, as expected, the combination of surgery at 2.5 years post referral for both the standard care and improved care pathways results in a median difference of 0%, 95% CrI [-1.9% to 2.1], with an error range indicating the randomness of sampling in the computations.

The analysis of return on surgical investment was also reassessed to determine the impact of postsurgical weight trajectory improvement. Repeat of the analysis in which patients in the surgical pathway experience standard care trajectories (rather than improved care trajectories) was performed (*Figure S6*). An overlay of the outcomes suggests that the time after surgery to achieve return on surgical investment trends to overall lower values when improved postsurgical weight trajectories are achieved compared to standard care trajectories after surgery.

The relative risk of comorbidity prevalence for surgical versus nonsurgical patients was determined for the case where, after surgery, patients achieve standard of care weight trajectory outcomes (*Figure S7*). Similar to the main text analysis, when the decision to undertake surgery or to drop out of the bariatric care program is delayed, the benefit of surgery in decreasing the risk of comorbidity prevalence is lowered. The poorer postsurgical trajectory outcomes, however, suggest a trend towards lesser reduction in risk of having any of the three comorbidities after surgery. Also, time of surgical delivery where the 95% CI includes 1.0 (indicative of no difference in relative risk) is earlier for each comorbidity than for the case where postsurgical trajectories are improved, meaning that if postsurgical trajectories remain at standard care levels, surgery must occur earlier to increase the likelihood of significant reduction in comorbidity risk.

6 References

1. Doumouras AG, Lee Y, Babe G, Gmora S, Tarride JE, Hong D, et al. The hidden cost of an extensive preoperative work-up: predictors of attrition after referral for bariatric surgery in a universal healthcare system. Surg Endosc. 2020;34(2):988-95.

2. Padwal RS, Rueda-Clausen CF, Sharma AM, Agborsangaya CB, Klarenbach S, Birch DW, et al. Weight loss and outcomes in wait-listed, medically managed, and surgically treated patients enrolled in a population-based Bariatric program: prospective cohort study. Med Care. 2014;52(3):208-15.

3. Diamant A, Cleghorn MC, Milner J, Sockalingam S, Okrainec A, Jackson TD, et al. Patient and operational factors affecting wait times in a bariatric surgery program in Toronto: a retrospective cohort study. CMAJ Open. 2015;3(3):E331-7.

4. Diamant A, Milner J, Cleghorn M, Sockalingam S, Okrainec A, Jackson TD, et al. Analysis of patient attrition in a publicly funded bariatric surgery program. J Am Coll Surg. 2014;219(5):1047-55.

5. Al Harakeh AB, Burkhamer KJ, Kallies KJ, Mathiason MA, Kothari SN. Natural history and metabolic consequences of morbid obesity for patients denied coverage for bariatric surgery. Surg Obes Relat Dis. 2010;6(6):591-6.

6. Davis JA, Saunders R. Comparison of Comorbidity Treatment and Costs Associated With Bariatric Surgery Among Adults With Obesity in Canada. JAMA Netw Open. 2020;3(1):e1919545.

7. Whitlock KA, Gill RS, Ali T, Shi X, Birch DW, Karmali S. Early Outcomes of Roux-en-Y Gastric Bypass in a Publically Funded Obesity Program. ISRN Obes. 2013;2013:296597.

8. Canadian Obesity Network. Report card on access to obesity treatment for adults in Canada 2017 Edmonton, AB: Canadian Obesity Network, Inc.; 2017 [updated July 18, 2017. Available from: https://obesitycanada.ca/publications/canadians-lack-access-obesity-treatments-support-report-card/.

9. Courcoulas AP, King WC, Belle SH, Berk P, Flum DR, Garcia L, et al. Seven-Year Weight Trajectories and Health Outcomes in the Longitudinal Assessment of Bariatric Surgery (LABS) Study. JAMA Surg. 2018;153(5):427-34.

10. Doumouras AG, Saleh F, Tarride JE, Hong D. A population-based analysis of the drivers of short-term costs after bariatric surgery within a publicly funded regionalized center of excellence system. Surg Obes Relat Dis. 2016;12(5):1023-31.

11. Luan WP, Leroux TC, Olsen C, Robb D, Skinner JS, Richard P. Variation in Bariatric Surgery Costs and Complication Rates in the Military Health System. Mil Med. 2019.

12. Saleh F, Doumouras AG, Gmora S, Anvari M, Hong D. Outcomes the Ontario Bariatric Network: a cohort study. CMAJ Open. 2016;4(3):E383-E9.

13. Gill RS, Majumdar SR, Rueda-Clausen CF, Apte S, Birch DW, Karmali S, et al. Comparative effectiveness and safety of gastric bypass, sleeve gastrectomy and adjustable gastric banding in a population-based bariatric program: prospective cohort study. Can J Surg. 2016;59(4):233-41.

14. Skulsky SL, Dang JT, Battiston A, Switzer NJ, Birch DW, Sharma AM, et al. Higher Edmonton Obesity Staging System scores are associated with complications following laparoscopic Roux-en-Y gastric bypass. Surg Endosc. 2019.

15. Doumouras AG, Albacete S, Mann A, Gmora S, Anvari M, Hong D. A Longitudinal Analysis of Wait Times for Bariatric Surgery in a Publicly Funded, Regionalized Bariatric Care System. Obes Surg. 2020;30(3):961-8.

16. Doumouras AG, Saleh F, Gmora S, Anvari M, Hong D. The value of surgical experience: excess costs associated with the Roux-en-Y gastric bypass learning curve. Surg Endosc. 2019;33(6):1944-51.

17. Larjani S, Spivak I, Hao Guo M, Aliarzadeh B, Wang W, Robinson S, et al. Preoperative predictors of adherence to multidisciplinary follow-up care postbariatric surgery. Surg Obes Relat Dis. 2016;12(2):350-6.

18. Elbahrawy A, Bougie A, Loiselle SE, Demyttenaere S, Court O, Andalib A. Medium to long-term outcomes of bariatric surgery in older adults with super obesity. Surg Obes Relat Dis. 2018;14(4):470-6.

19. Rousseau C, Jean S, Gamache P, Lebel S, Mac-Way F, Biertho L, et al. Change in fracture risk and fracture pattern after bariatric surgery: nested case-control study. BMJ. 2016;354:i3794.

20. McIsaac M, Kaban G, Clay A, Berry W, Prasad B. Long-Term Impact of Bariatric Surgery on Renal Outcomes at a Community-Based Publicly Funded Bariatric Program: The Regina Bariatric Study. Can J Kidney Health Dis. 2019;6:2054358119884903.

21. Lemus R, Karni D, Hong D, Gmora S, Breau R, Anvari M. The impact of bariatric surgery on insulin-treated type 2 diabetes patients. Surg Endosc. 2018;32(2):990-1001.

22. Strain GW, Saif T, Gagner M, Rossidis M, Dakin G, Pomp A. Cross-sectional review of effects of laparoscopic sleeve gastrectomy at 1, 3, and 5 years. Surg Obes Relat Dis. 2011;7(6):714-9.

23. Ladak F, Dang JT, Switzer NJ, Mocanu V, Birch DW, Karmali S. Rates of reoperation and nonoperative intervention within 30 days of bariatric surgery. Surg Obes Relat Dis. 2019;15(3):431-40.

24. Anvari M, Lemus R, Breau R. A Landscape of Bariatric Surgery in Canada: For the Treatment of Obesity, Type 2 Diabetes and Other Comorbidities in Adults. Can J Diabetes. 2018;42(5):560-7.

7 Tables

Table S1 Summary of baseline comorbidity prevalence in Canadian bariatric surgery patients

Province	Source	Surgery	N	Diabetes	Hypertension	Dyslipidemia	BMI (kg/m^2)	Age (years)
Alberta	Gill et al.,(13) 2016	RYGB	51	45.1%	64.7%	62.7%	48.8 ± 6.9	41.9 ± 8.4
	Skulsky et al.,(14) 2019	RYGB	378	33.3%	44.4%	31.5%	45.6 ± 6.3	45.6 ± 9.9
	Padwal et al.,(2) 2014[†]	RYGB (51/150), SG (51/150), AGB (48/150)	150	50.0%	66.0%	59.3%	49.4 ± 8.2	43.6 ± 9.2
	Whitlock et al.,(7) 2013	RYGB	293	28.0%	49.5%	NR	55.3 ± 10.0	41.6 ± 9.3
	Weighted provincial average			35.1%	51.0%	35.2%		
Ontario	Doumouras et al.,(15) 2020	Not specified	18,854	39.5%	53.3%	NR	48.5 ± 9.2	NR
	Doumouras et al.,(1) 2020	Not specified	17,703	38.5%	55.3%	NR	48.1 ± 6.4	44.8 ± 8.5
	Doumouras et al.,(16) 2019	Not specified	11,684	28.6%	27.1%	NR	NR	44.6 ± 10.4
	Doumouras et al.,(10) 2016	RYGB (91.7%), SG (8.3%)	5,007	29.6%	27.2%	3.5%	NR	NR
	Larjani et al.,(17) 2016	RYGB (91.8%), SG (8.2%)	388	43.8%	50.5%	NR	44.9 ± 11.1	49.4 ± 8.2
	Saleh et al.,(12) 2016	RYGB (91.7%), SG (8.3%)	4,591	29.1%	26.3%	3.3%	NR	44.4 ± 10.3
	Weighted provincial average			35.4%	44.2%	3.4%		

Province	Source	Surgery	N	Diabetes	Hypertension	Dyslipidemia	BMI (kg/m^2)	Age (years)
Quebec	Elbahrawy et al.,(18) 2017	RYGB (16%), SG (74%), BPD (8%)	107	60.0%	78.0%	NR	51.7 ± 8.1	NR
	Rousseau et al.,(19) 2016	Not specified	12,676	31.2%	29.6%	NR	NR	42.6 ± 11.0
	Weighted provincial average			31.4%	30.0%			
Saskatchewan	McIsaac et al.,(20) 2019	RYGB (80.7%), SG (19.3%)	471	27.8%	NR	NR	47.7 ± 7.8	46.0 ± 10.0

[†]Note that the prevalences shown for the study of Padwal et al.,(2) 2014 are those used in the present study and correspond to the baseline reported for the waitlisted group; the prevalence of comorbidities differed for the surgical group shown, who underwent different types of bariatric surgery as indicated. The weighted average calculated per province treats each study independently, however it is assumed there will considerable overlap in Ontario for example, since patients are drawn from the same database. AGB, adjustable gastric band; BPD, biliopancreatic diversion; BMI, body mass index; NR, not reported; RYGB, Roux-en-Y gastric bypass; SG, sleeve gastrectomy.

Table S2 Wait times for bariatric surgery in Canada by province

	Wait from referral to consult			Wait from consult to surgery			Overall wait time	
Province	Minimum (months)	Maximum (months)	Midpoint (months)	Minimum (months)	Maximum (months)	Midpoint (months)	Total (months)	Total (years)
Newfoundland and Labrador	24	24	24	12	12	12	36	3.00
Nova Scotia	60	60	60	6	6	6	66	5.50
New Brunswick	36	48	42	12	12	12	54	4.50
Quebec	24	24	24	6	12	9	33	2.75
Ontario	12	24	18	6	12	9	27	2.25
Manitoba	48	48	48	12	12	12	60	5.00
Saskatchewan	24	24	24	6	6	6	30	2.50
Alberta	18	24	21	12	24	18	39	3.25
British Colombia	24	24	24	6	12	9	33	2.75
Canadian average								3.50

Wait times were provided in months as minima and maxima from a 2017 report.(8) The midpoint of each range was calculated and summed to get the overall wait time, then converted into years. At the time of the report, bariatric surgeries were not performed in Prince Edward Island.

Table S3 Weight loss outcomes after Roux-en-Y gastric bypass surgery in Canada

Source	Follow up time (months)	Total weight loss
Skulsky et al.,(14) 2019	12	26.3% ± 9.2%
Lemus et al.,(21) 2018	3	19.7% ± 5.5%
	6	26.6% ± 7.3%
	12	31.3% ± 8.2%
	24	31.2% ± 9.5%
	36	29.3% ± 9.7%
Elbahrawy et al.,(18) 2017	24	30.3% ± 11.0%
Gill et al.,(13) 2016	6	19.2% ± 1.9%
	12	24.8% ± 2.7%
	18	26.0% ± 3.3%
	24	26.1% ± 3.4%
Strain et al.,(22)2014	19.4	33.5% ± 12.1%

Results are shown specific to Roux-en-Y gastric bypass surgery; studies of general bariatric surgery, or where outcomes could not specifically be attributed to Roux-en-Y gastric bypass were not included. Studies reporting results after a mean or median follow-up time are included, and these consist of a single time point. Values are reported as total weight loss and the low and high boundaries of the corresponding 95% confidence interval

Table S4 Population proportions of postsurgical weight loss trajectories

Trajectory group	Courcoulas et al.,(9) 2018	Present study standard care	Present study improved care
G1	4.8%	0.0%	0.0%
G2	21.6%	28.6%	0.0%
G3	27.8%	36.7%	41.2%
G4	26.3%	34.7%	39.0%
G5	6.1%	0.0%	0.0%
G6	13.3%	0.0%	19.8%

The proportions of patients belonging to each trajectory group ranging from group 1 (G1, lowest overall weight loss) to group 6 (G6, highest overall weight loss) are shown for the original study(9) of Courcoulas et al., 2018. Overlaying data from Canadian RYGB studies (main text Figure 1 and supplement Table S3) suggests that trajectory groups 2 and 3 are most representative of Canadian outcomes. In the improved care scenario, patients are redistributed among the top 2 trajectory groups (groups 4, and 6), exclusive of group5.

Table S5 *Frequency and costs for individual complications after Roux-en-Y gastric bypass surgery in Ontario, 2009-2011*

Complication	N	%	Adjusted cost	95% CI low	95% CI high	Impact (freq * cost)	Uncertainty
Anastomotic leaks	118	2.6%	$24,397	$20,688	$28,106	$634	$112
Minor infectious	185	4.0%	$404	-$172	$980	$16	$23
Hemorrhage with OR	25	0.5%	$12,350	$9,526	$15,174	$62	$19
Minor hemorrhage	176	3.8%	$2,048	$1,558	$2,537	$78	$19
SBO with OR	28	0.6%	$13,541	$10,302	$16,779	$81	$25
Minor SBO	22	0.5%	$402	-$950	$1,754	$2	$7
Other reoperations	52	1.1%	$9,126	$7,503	$10,749	$100	$23
Cardiac arrest	15	0.3%	$4,635	$1,675	$7,596	$14	$10
Thrombosis	22	0.5%	$3,939	$2,021	($5,858)	$20	$20
Renal failure	31	0.7%	$7,839	$5,103	$10,575	$55	$21
Respiratory failure/infection	38	0.8%	$19,465	$11,007	$27,924	$156	$72
Other minor complications	86	1.9%	$1,246	$508	$1,983	$24	$14
Overall complication cost						$1,242	$147
Readmissions	N	%	Costs per readmission				
0	4,308	93.9%	$2,213	$1,850	$2,577	$0	$0
1	246	5.4%	$2,213	$1,850	$2,577	$120	$21
2	32	0.7%	$2,213	$1,850	$2,577	$31	$4
3	5	0.1%	$2,213	$1,850	$2,577	$7	$1
Readmission costs						$157	$21
Total overall burden						$1,399	$148

List of individual complication frequency and associated costs in Ontario from the study of Doumouras et al., (10) 2016 of 4.591 gastric bypass surgeries that occurred between 2009 and 2011. The average impact of each complication is calculated as the frequency times the adjusted cost and uncertainty is determined from the reported 95% confidence intervals. The overall cost of complications is the sum of the individual impacts and the uncertainty is the addition in quadrature of the uncertainty for each complication impact cost according to error of propagation principles. OR, [requiring] operation; SBO, small bowel obstruction.

Table S6   Determination of increase in Roux-en-Y gastric bypass costs due to complications

Year	RYGB cost (mean ± SD)
2009-2010	$13,253 ± $47,539
2010-2011	$10,656 ± $22,200
2011-2012	$9,359 ± $12,805
Average (mean ± SEM)	$11,089 ± $10,394
Note that the above average will include costs of complications.<br>Subtract complication costs to determine cost of an uncomplicated procedure	
Complication burden (Table S5)	$1,399 ± $148
Cost of uncomplicated procedure	$9,691 ± $10,395
Percent increase<br>(complication costs/cost of uncomplicated procedure)	14.4% ± 15.6%

Calculation of percent increase in costs for Roux-en-Y gastric bypass surgery. SEM, standard error of the mean.

Table S7 Incidence of complications after bariatric surgery in Canada and North America

Source	Setting	Surgery	N	Period	Rate	Note
Luan et al.,(11) 2020	United States	RYGB, SG, BPD, AGB	1,277	Index admission	15.5%	
Ladak et al.,(23) 2019	North America	RYGB	69,411	30 days	5.5%	Quality improvement database for all North America, 2015-2016
Skulsky et al.,(14) 2019	Alberta	RYGB	378	1 year	9.3%	Major complications
Anvari et al.,(24) 2017	Canada	Not specified	NR	Perioperative	5.0%	Cited from report to year ending 2013 describing decreasing trend from 8.2%
Elbahrawy et al.,(18) 2017	Canada	RYGB, SG, BPD, revision	104	All	15.4%	Population was older (>= 60 y) and included cohort of super-obese (BMI > 50 kg/m^2)
				Early	6.7%	
				Late	8.7%	
Doumouras et al.,(10) 2016	Ontario	RYGB, SG	5,007	30 days	8.0%	Any complication, most did not require readmission (54 not readmitted of 403 reported as having "any complication")
Gill et al.,(13) 2016	Alberta	RYGB	51	2 years	19.6%	Surgery-related adverse events within 2 years of surgery but no stratification for early complications (within 30 days)
Saleh et al.,(12) 2016	Ontario	RYGB	4,591	30 days	11.9%	
Present study	Canada	RYGB	NA	NA	10.2%	Mean of reported rates

Reported rates of complications related to bariatric surgery inclusive of Canadian data. The mean complication rate determined for the present study is the arithmetic mean of the other given rates; only the complication rate for “all” in the Elbahrawy et al.,(18) 2016 study was used and the results of Gill et al., 2016 were not included in the mean since the time period of consideration was long at 2 years postsurgery. A simple arithmetic mean, rather than a mean weighted by number of patients, was used to allow each study to contribute equally; the result would otherwise be dominated by the North American database analysis of Ladak et al.,(23) 2019 the result of which may not be representative of the Canadian experience. “Major complications” defined according to Clavien-Dindo scale ≥ IIIa as the first level requiring “endoscopic, surgical, or radiologic intervention”.(14) AGB, adjustable gastric band; BPD, Biliopancreatic diversion; NA, not applicable; NR, not reported; RYGB, Roux-en-Y gastric bypass; SG, sleeve gastrectomy.

Table S8 Percentage change in total costs by varying wait time in standard and improved care pathways with improvement in postsurgical weight trajectories

	Improved care pathway time referral to surgery				
	6 months	1.0 year	1.5 years	2.0 years	2.5 years
Standard care pathway time referral to surgery	% change, Median [95% CrI]	% change, Median [95% CrI]	% change, Median [95% CrI]	% change, Median [95% CrI]	% change, Median [95% CrI]
2.5 years	-37.5% [-46.0% to -28.0%]	-29.5% [-35.9% to -23.0%]	-22.8% [-27.8% to -18.1%]	-16.8% [-20.7% to -12.6%]	-11.8% [-15.0% to -7.4%]
3.0 years	-39.6% [-48.6% to -29.4%]	-31.9% [-38.7% to -24.5%]	-25.4% [-31.0% to -19.9%]	-19.5% [-24.0% to -15.2%]	-14.6% [-18.1% to -10.9%]
3.5 years	-41.1% [-50.4% to -30.7%]	-33.7% [-41.1% to -25.7%]	-27.4% [-33.6% to -20.8%]	-21.7% [-26.8% to -16.4%]	-16.8% [-20.9% to -12.8%]
4.0 years	-42.5% [-52.4% to -31.9%]	-35.2% [-43.4% to -26.3%]	-29.1% [-36.0% to -21.7%]	-23.5% [-29.4% to -17.5%]	-18.8% [-23.6% to -14.0%]
4.5 years	-43.6% [-53.8% to -32.8%]	-36.4% [-45.1% to -27.0%]	-30.3% [-38.0% to -22.3%]	-24.9% [-31.5% to -18.1%]	-20.3% [-25.8% to -14.7%]

The percentage change in total costs are shown for varying combinations of surgical delivery time in the improved pathway (6 months to 2.5 years as indicated at the top) versus surgical delivery in the standard care pathway (2.5 years to 4.5 years as indicated at the side). The improved pathway also includes improved postsurgical weight loss trajectories. Change is for improved path costs relative to standard path costs thus negative values indicate cost reductions for the improved care pathway. Note that surgery at the same time (2.5 years, upper left cell) in both pathways has cost reductions indicated due to the separate contribution of the postsurgical weight loss improvement. CrI, credibility interval.

Table S9 Percentage change in total costs by varying wait time in standard and improved care pathways, both paths with standard care postsurgical weight trajectories

	Improved care pathway time referral to surgery				
	6 months	1.0 year	1.5 years	2.0 years	2.5 years
Standard care pathway time referral to surgery	% change, Median [95% CrI]	% change, Median [95% CrI]	% change, Median [95% CrI]	% change, Median [95% CrI]	% change, Median [95% CrI]
2.5 years	-21.1% [-30.4% to -9.3%]	-14.1% [-20.7% to -6.0%]	-8.5% [-13.4% to -3.8%]	-3.7% [-7.0% to -1.3%]	0.0% [-1.9% to 2.1%]
3.0 years	-23.7% [-33.7% to -11.4%]	-17.0% [-24.5% to -7.9%]	-11.6% [-17.5% to -5.6%]	-7.0% [-11.2% to -3.3%]	-3.2% [-6.1% to -1.3%]
3.5 years	-25.6% [-36.2% to -12.9%]	-19.1% [-27.5% to -9.5%]	-13.8% [-20.8% to -7.0%]	-9.4% [-14.9% to -4.4%]	-5.8% [-9.8% to -2.5%]
4.0 years	-27.4% [-38.7% to -14.6%]	-21.0% [-30.2% to -11.1%]	-15.9% [-24.0% to -7.9%]	-11.5% [-18.1% to -5.4%]	-8.0% [-13.1% to -3.4%]
4.5 years	-28.6% [-40.8% to -16.1%]	-22.4% [-32.5% to -12.0%]	-17.4% [-26.4% to -8.6%]	-13.1% [-20.7% to -5.8%]	-9.6% [-15.9% to -3.9%]

The percentage change in total costs are shown for varying combinations of surgical delivery time in the improved pathway (6 months to 2.5 years as indicated at the top) versus surgical delivery in the standard care pathway (2.5 years to 4.5 years as indicated at the side). In this analysis, both the improved and standard care pathway have the same (standard care) postsurgical weight loss outcomes. Change is for improved path costs relative to standard path costs thus negative values indicate cost reductions for the improved care pathway. Note that surgery at the same time (2.5 years, upper left cell) in both pathways is associated with a median difference of 0% as expected, since both care pathways have the same postsurgical weight loss outcomes. CrI, credibility interval.

8 Figures and captions

Figure S1 Bariatric surgery dropout models in the Canadian setting

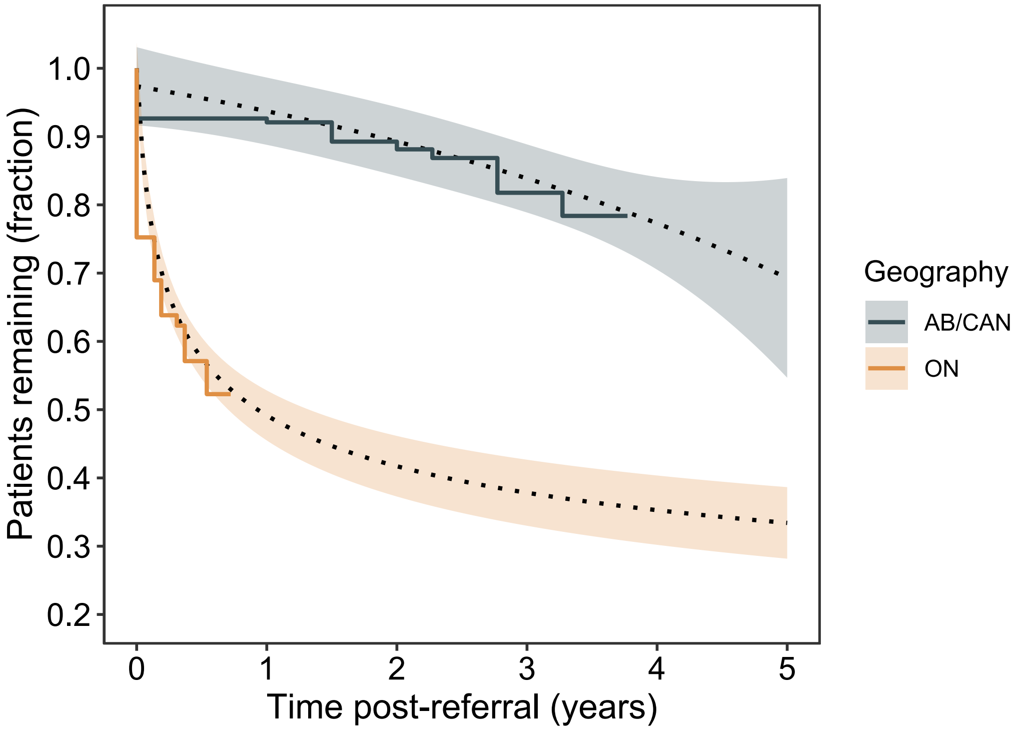

Data are shown for patient dropout according to studies in Canada. Raw data are shown as solid stepped lines, broken lines indicate modeled fit and shaded regions correspond to 95% confidence intervals. The data Padwal et al.,(2) were obtained in the Alberta setting and these were used to represent expected dropout rates on average across Canada (AB/CAN). Rates for Ontario (ON) were determined from the studies of Diamant et al.,(4) 2014 which reported patient attrition at the various stages of the Ontario bariatric care pathway and a subsequent study by Diamant et al.,(3) 2015 that reported the average time between each stage. AB/CAN, Alberta/Canada; ON, Ontario.

Figure S2 Annual and cumulative difference in patient-years of comorbidity treatment after Roux-en-Y gastric bypass surgery

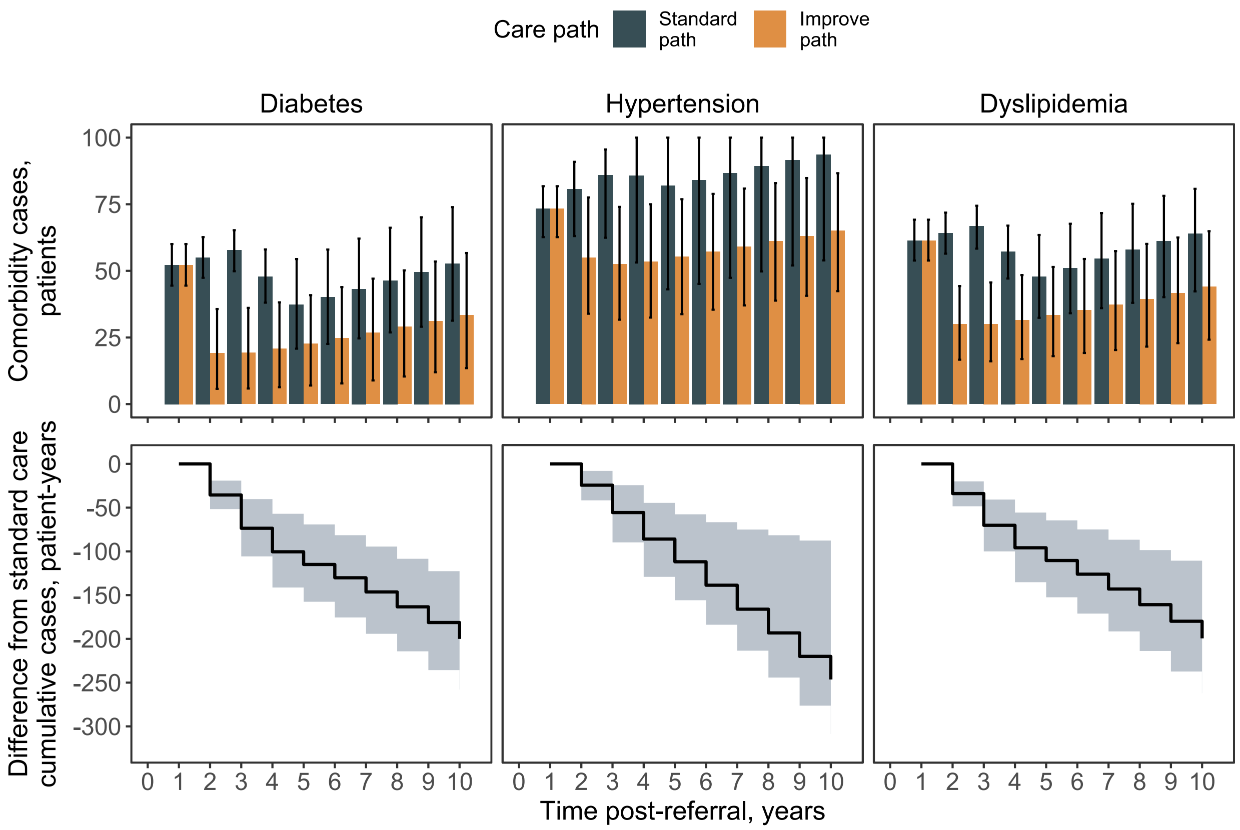

Patient-years of treatment for the three comorbidities assessed in the present study are shown by year (top). The difference (bottom) in the base case indicates the cumulative annual change in prevalence between the standard care pathway and the improved care pathway for which surgery has been brought forward (1 year versus 3.5 years) and after surgery, the weight trajectory mixture of patients corresponds to better weight loss outcomes. Values displayed (bars, lines) correspond to medians and uncertainty (error bars, shaded band) corresponds to 95% CrIs.

Figure S3 Sensitivity analysis, total costs varying time of surgery in standard and improved care pathways, inclusive of postsurgical weight trajectory improvement

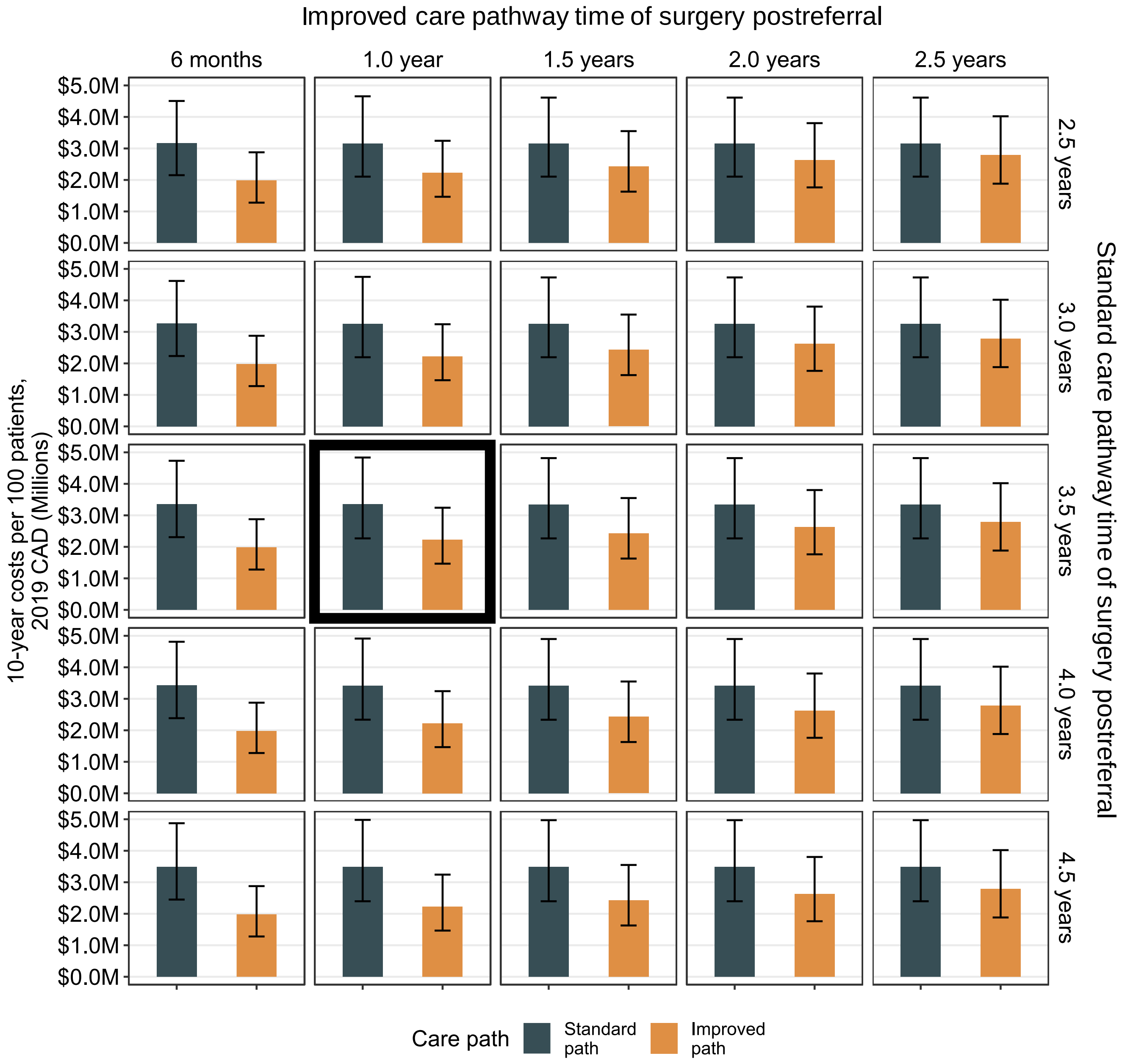

Total costs over the 10-year time horizon for 100-patient cohorts following the standard care pathway or the improved care pathway according to time of surgery delivery. Note that post surgery, patients in the standard care pathway follow standard care outcomes of weight trajectory, while those in the improved pathway exhibit improved weight trajectories of greater weight loss. The combination with the heavy black outline of 1 year surgery in the improved path and 3.5 years in the standard care path corresponds to the base case analysis. CAD, Canadian dollars.

Figure S4 Sensitivity analysis of impact of time of surgical delivery on postsurgical weight trajectory improvement

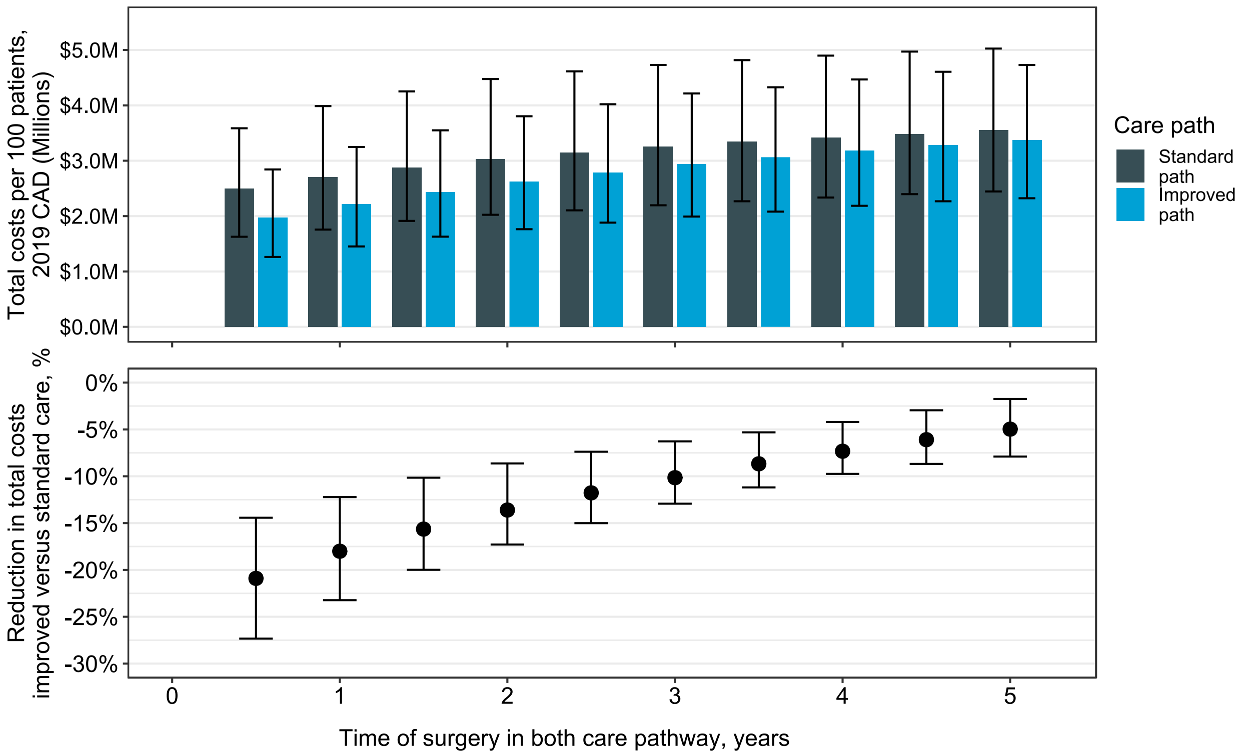

Total costs over the 10-year time horizon for 100-patient cohorts are shown in which patients in both pathways receive surgery at the same time post referral, but patients in the improved path experience increased weight loss (improved postsurgical trajectory) while those in the standard care path experience the standard care outcomes. CAD, Canadian dollars.

Figure S5 Sensitivity analysis, total costs varying time of surgery in standard and improved care pathways, both care pathways with standard care postsurgical weight trajectory

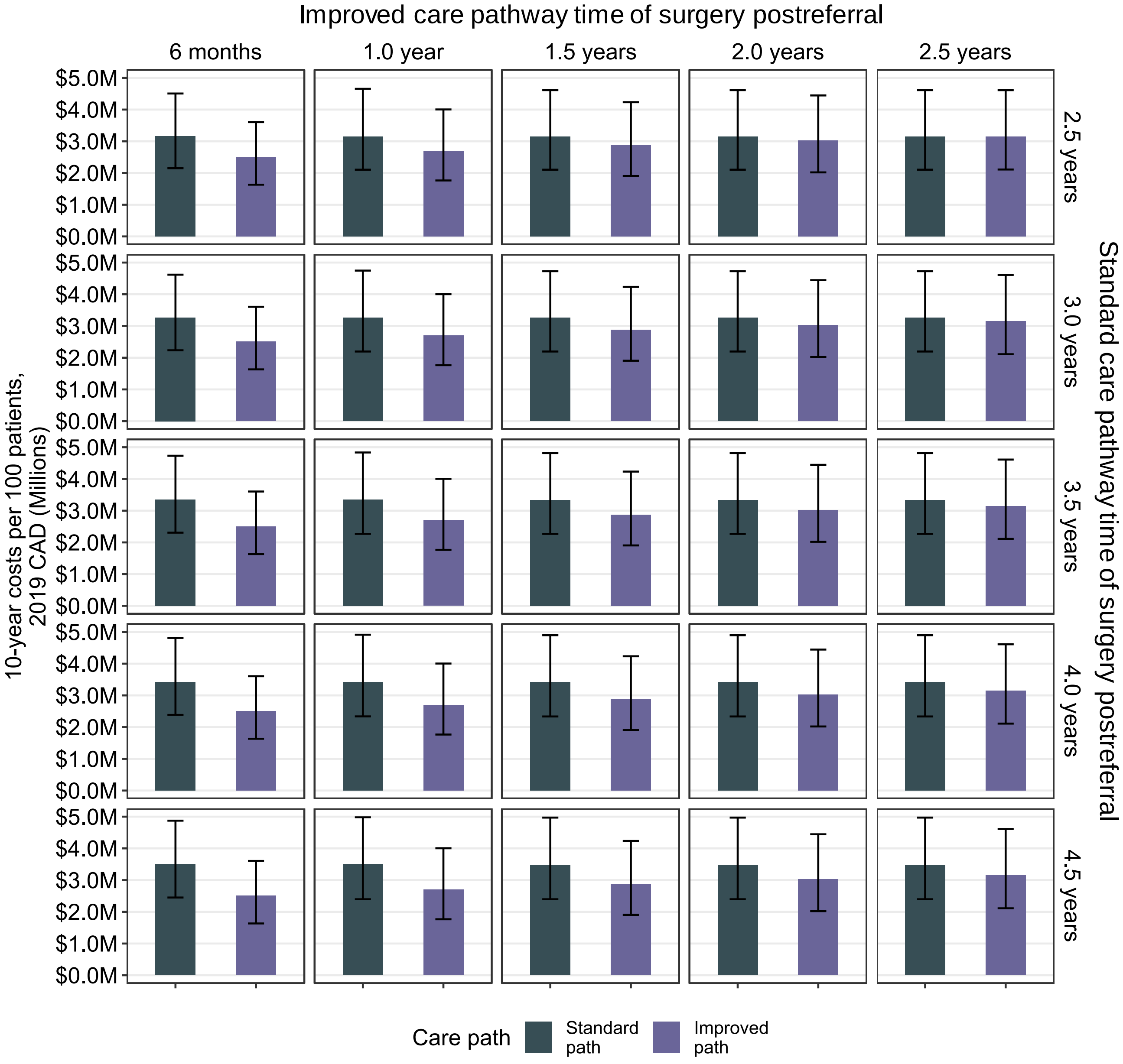

Total costs over the 10-year time horizon for 100-patient cohorts following the standard care pathway or the improved care pathway according to time of surgery delivery. Note that post surgery, patients in the standard care pathway follow standard care outcomes of weight trajectory, while those in the improved pathway exhibit improved weight trajectories of greater weight loss. CAD, Canadian dollars.

Figure S6 Sensitivity analysis of time post surgery to achieve return on surgical investment with standard care postsurgical weight trajectories

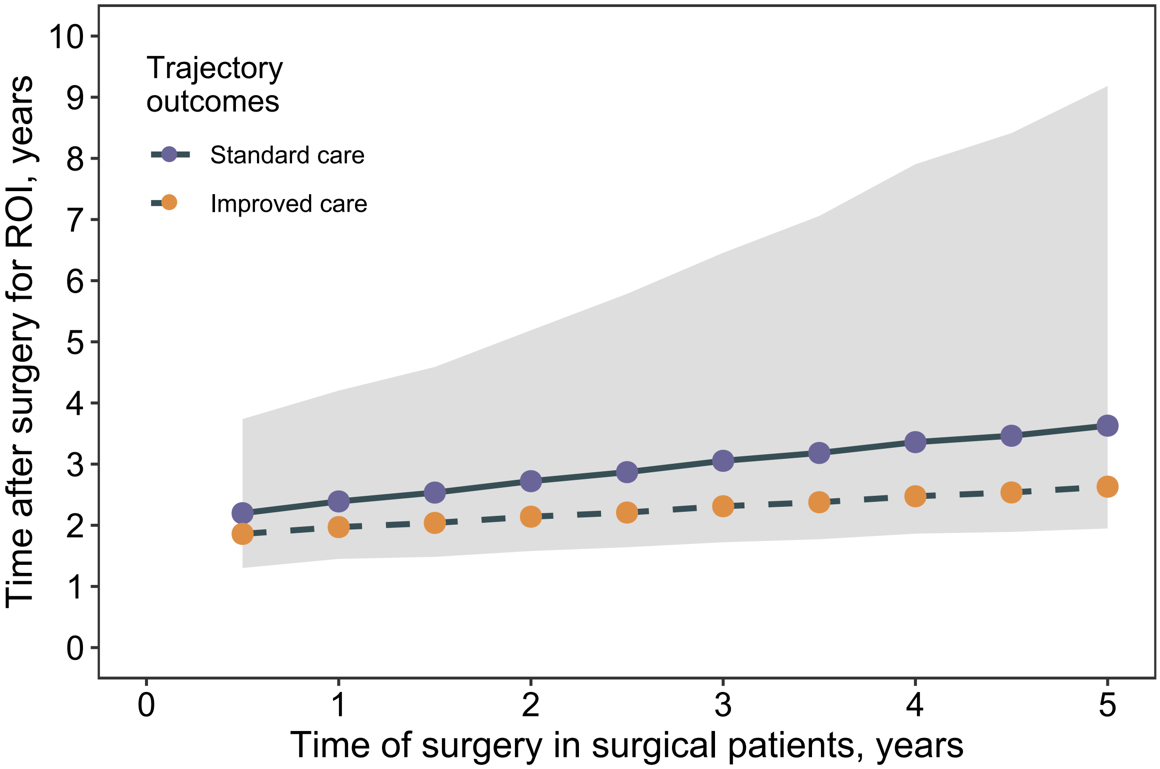

A sensitivity analysis corresponding to Figure 4 of the main text is shown, comparing surgical with nonsurgical patients. Patients in the surgical care pathway achieve standard care postsurgical weight trajectory outcomes. For comparison, the results from the main text (Figure 4) are superimposed as a broken line. The shaded region corresponds to the 95% CrI around the line of standard care post-RYGB trajectory outcomes. ROI, return on (surgical) investment.

Figure S7 Sensitivity analysis of relative risk of comorbidity prevalence for surgical versus nonsurgical patients

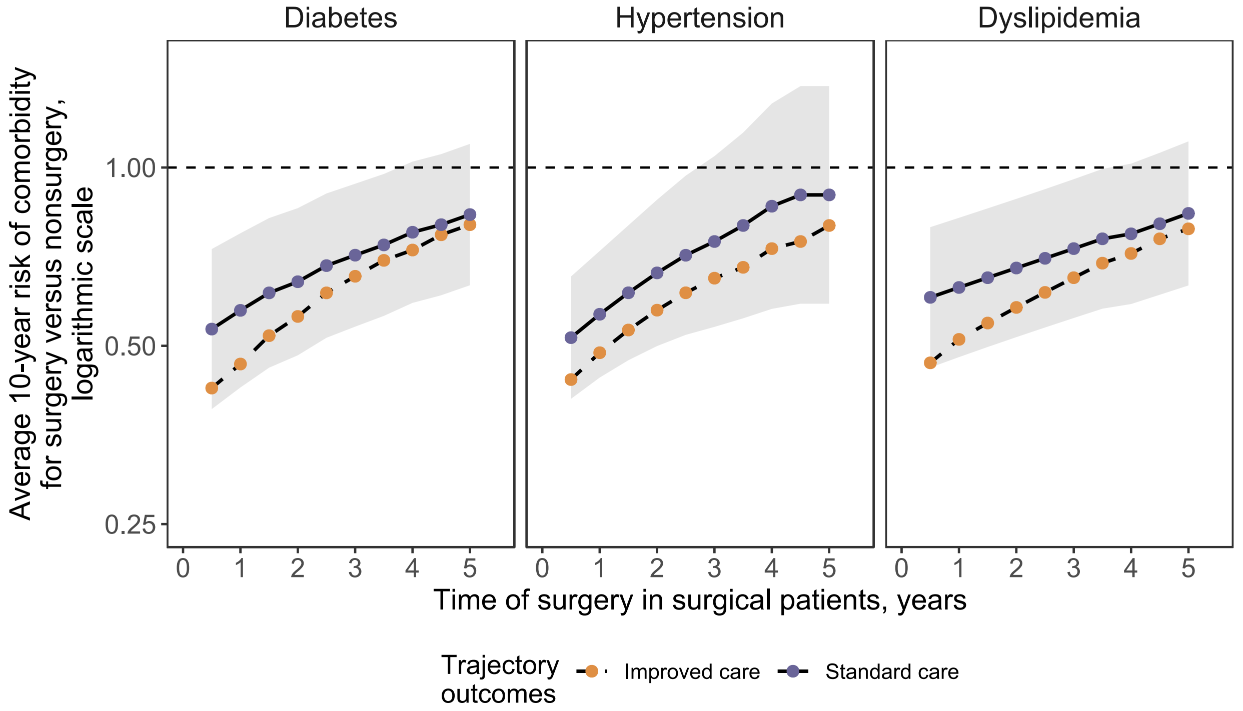

A sensitivity analysis corresponding to Figure 5 of the main text is shown, comparing surgical with nonsurgical patients. Patients in the surgical care pathway achieve standard care postsurgical weight trajectory outcomes and plotted are the relative risk of having the indicated comorbidity versus nonsurgical patients. For comparison, the results from the main text (Figure 5) where patients achieve the improved weight loss trajectories after surgery are superimposed as a broken line. The shaded regions correspond to the 95% CI (confidence intervals as the risk ratios were determined using normal distribution parametric statistics) about the risk ratios for the line of standard care post-RYGB outcomes.
