# Supplementary figures and images for: Earlier Provision of Gastric Bypass Surgery in Canada Enhances Surgical Benefit and Leads to Cost and Comorbidity Reduction
Source: Front Public Health. 2020 Sep 30;8:515. doi: 10.3389/fpubh.2020.00515 (PMC7554569; doi:10.3389/fpubh.2020.00515)

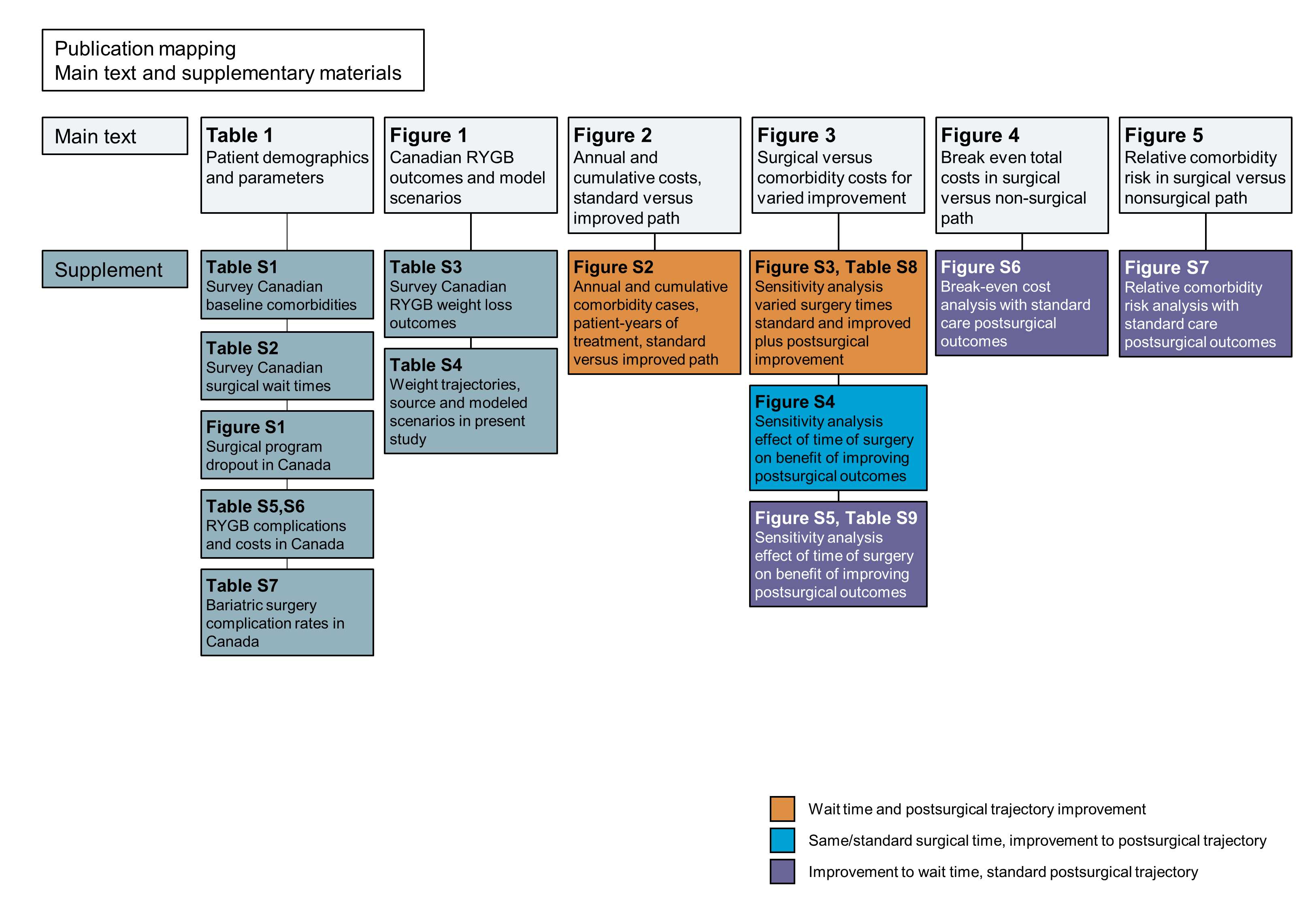

Supplement: Supplementary file 2 [file Image_1.TIF]

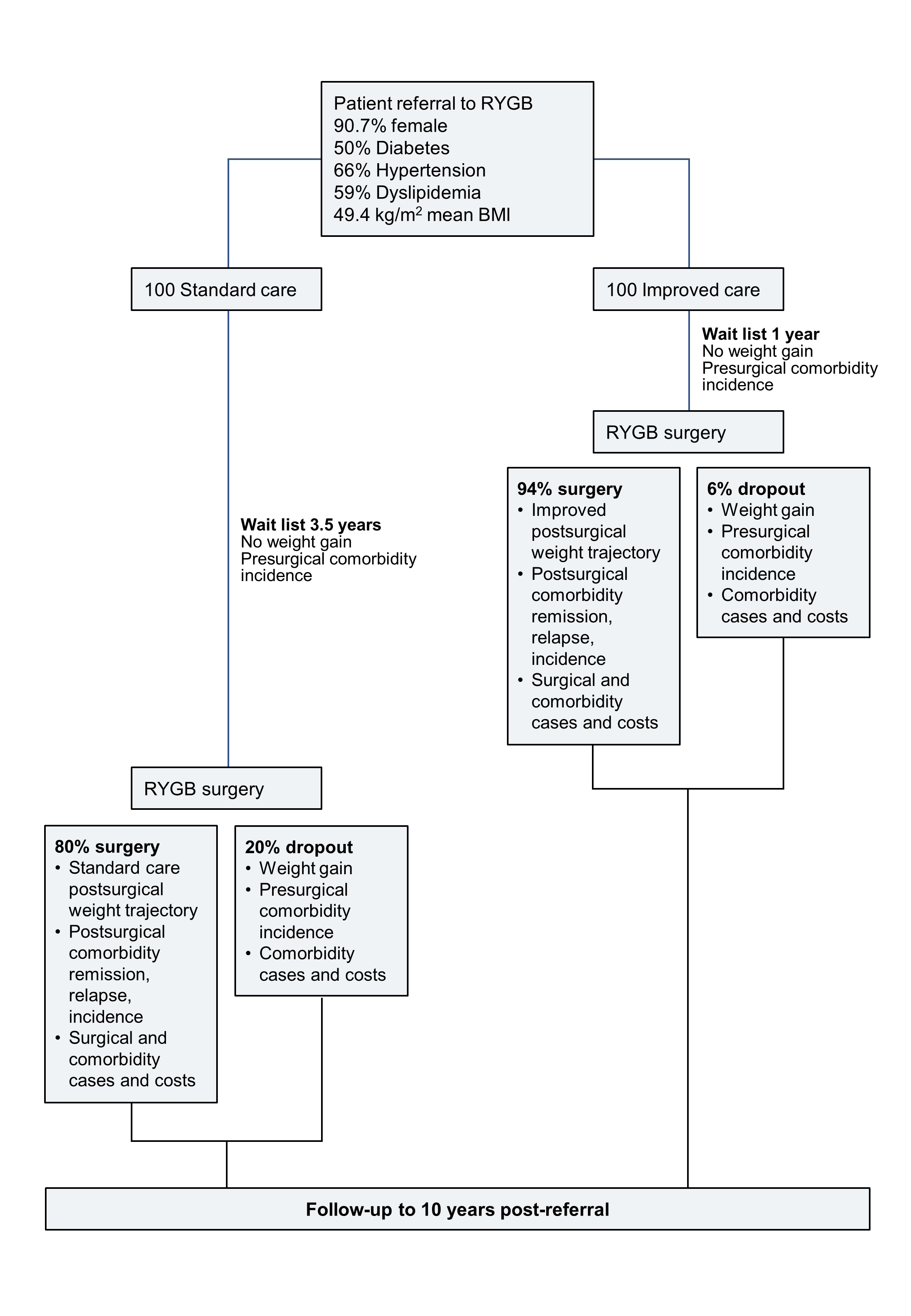

Supplement: Supplementary file 3 [file Image_2.TIF]
